# Phosphomevalonate Kinase Controls *β*‐Catenin Signaling via the Metabolite 5‐Diphosphomevalonate

**DOI:** 10.1002/advs.202204909

**Published:** 2023-02-21

**Authors:** Zhiqiang Chen, Xinyi Zhou, Xiaojun Zhou, Yi Tang, Mingzhu Lu, Jianhong Zhao, Chenhui Tian, Mingzhi Wu, Yanliang Liu, Edward V. Prochownik, Fubing Wang, Youjun Li

**Affiliations:** ^1^ Hubei Key Laboratory of Cell Homeostasis College of Life Sciences Frontier Science Center for Immunology and Metabolism TaiKang Center for Life and Medical Sciences Wuhan University Wuhan 430072 P. R. China; ^2^ Medical Research Institute Zhongnan Hospital of Wuhan University Wuhan University Wuhan 430071 P. R. China; ^3^ Department of Gastrointestinal Surgery Renmin Hospital of Wuhan University Wuhan 430060 P. R. China; ^4^ Division of Hematology/Oncology Children's Hospital of Pittsburgh of UPMC Department of Microbiology and Molecular Genetics Pittsburgh Liver Research Center and Hillman Cancer Center of UPMC University of Pittsburgh Medical Center Pittsburgh PA 15224 USA; ^5^ Department of Laboratory Medicine and Center for Single‐Cell Omics and Tumor Liquid Biopsy Zhongnan Hospital of Wuhan University Wuhan 430071 P. R. China; ^6^ Wuhan Research Center for Infectious Diseases and Cancer Chinese Academy of Medical Sciences Wuhan 430071 P. R. China

**Keywords:** *β*‐catenin, casein kinase I*α*, hepatocellular carcinoma, mevalonate 5‐diphosphate, phosphomevalonate kinase

## Abstract

*β*‐catenin signaling is abnormally activated in cancer. Here, this work screens the mevalonate metabolic pathway enzyme PMVK to stabilize *β*‐catenin signaling using a human genome‐wide library. On the one hand, PMVK‐produced MVA‐5PP competitively binds to CKI*α* to prevent *β*‐catenin Ser45 phosphorylation and degradation. On the other hand, PMVK functions as a protein kinase to directly phosphorylate *β*‐catenin Ser184 to increase its protein nuclear localization. This synergistic effect of PMVK and MVA‐5PP together promotes *β*‐catenin signaling. In addition, PMVK deletion impairs mouse embryonic development and causes embryonic lethal. PMVK deficiency in liver tissue alleviates DEN/CCl_4_‐induced hepatocarcinogenesis. Finally, the small molecule inhibitor of PMVK, PMVKi5, is developed and PMVKi5 inhibits carcinogenesis of liver and colorectal tissues. These findings reveal a non‐canonical function of a key metabolic enzyme PMVK and a novel link between the mevalonate pathway and *β*‐catenin signaling in carcinogenesis providing a new target for clinical cancer therapy.

## Graphical abstract

The mevalonate pathway kinase PMVK stabilizes *β*‐catenin via the PMVK‐generated intermediate MVA‐5PP. MVA‐5PP blocks *β*‐catenin phosphorylation at Ser45 by inhibiting the CKI*α* kinase. This stabilizes *β*‐catenin by preventing its cytoplasmic sequestration and proteolysis. Concurrently, PMVK also phosphorylates *β*‐catenin at Ser184, which facilitates its nuclear translocation. PMVK thus represents a novel therapeutic target in cancers that rely on aberrant *β*‐catenin signaling.

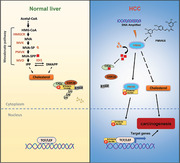

## 1 Introduction

Hepatocellular carcinoma (HCC) is the second leading cause of cancer‐related deaths worldwide.^[^ ^1^ ^]^ Although the current treatment of HCC mainly includes surgical resection, tumor ablation and chemotherapy, long‐term survival remains dismal.^[^ ^2^ ^]^ Metabolic reprogramming, considered one of the hallmarks of cancer,^[^ ^3^ ^]^ can involve changes in lipid metabolism, which supplies membrane components needed to support tumor cell proliferation. The targeting of lipid metabolism has thus been examined as a promising treatment option.^[^ ^4^ ^]^

The mevalonate anabolic pathway provides metabolites for multiple cellular processes in eukaryotes, archaea, and bacteria.^[^ ^5^ ^]^ Mevalonate, which is produced from acetoacetyl‐CoA, is converted to sterol isoprenoids such as cholesterol, which is an indispensable precursor of bile acids, lipoproteins, and steroid hormones. Mevalonate is also used in the synthesis of other hydrophobic molecules including nonsterol isoprenoids.^[^ ^4^, ^5^ ^]^ The role of these mevalonate pathways in tumorigenesis and progression has been extensively described.^[^ ^5^, ^6^ ^]^ In addition to the final metabolite cholesterol, mevalonate kinase promotes tumor growth by stabilizing mutant P53 protein levels through the metabolite mevalonate 5‐phosphate (MVA‐5P).^[^ ^7^ ^]^ Geranylgeranyl diphosphate (GGPP) and farnesyl pyrophosphate (FPP) can be used as geranylgeranylation and farnesylation substrates in post‐translational modifications of proteins generically known as protein prenylation. Such post‐translational modifications are required for the proper sub‐cellular targeting and activation of many oncogenic proteins, including some members of the RAS family.^[^ ^8^ ^]^

Phosphomevalonate kinase (PMVK) catalyzes the cation‐dependent reaction of MVA‐5P with ATP to form mevalonate 5‐diphosphate (MVA‐5PP) and ADP and represents a key step in the mevalonate pathway for isoprenoid/sterol biosynthesis and protein prenylation. Intermediates of this network play important roles in the post‐translational modification of numerous proteins involved in inter‐ and intra‐cellular signaling. Although the mevalonate pathway has been implicated in various aspects of cancer development and progression, the role(s) of PMVK remains unknown.^[^ ^9^ ^]^

The highly conserved Wnt‐*β*‐catenin signaling pathway regulates embryonic development, hepatobiliary development and homeostasis, and the repair response to liver injury.^[^ ^10^ ^]^ Abnormal Wnt‐*β*‐catenin signaling is also closely related to the development of many cancers.^[^ ^11^ ^]^ In the absence of active WNT signaling, cytoplasmic *β*‐catenin is rapidly degraded by the “APC complex” that includes the adenomatous polyposis coli (APC) tumor suppressor protein, Axin, glycogen synthase kinase (GSK)‐3*β*, casein kinase I*α* (CKI*α*) and the E3‐ubiquitin ligase *β*‐TrCP. *β*‐catenin's phosphorylation by GSK‐3 and CKI*α* within the APC Complex facilitates *β*‐TrCP‐mediated *β*‐catenin ubiquitination and proteasomal degradation.^[^ ^12^ ^]^ Genetic alteration in the core components of Wnt/*β*‐catenin signaling, most notably *β*‐catenin itself, lead to WNT‐independent signaling, which can drive tumorigenesis.^[^ ^12^, ^13^ ^]^ Mutations of the *CTNNB1* gene, which encodes *β*‐catenin occur in most cases of HCC.^[^ ^14^ ^]^ Wnt‐*β*‐catenin activation can increase fatty acid utilization to promote tumor progression and mutant forms of *β*‐catenin can increase glutamine levels in tumor cells to drive anaplerotic oxidative metabolism.^[^ ^15^ ^]^ The accumulation of cholesterol leads to reduced expression of squalene epoxidase which disrupts the GSK3*β* and p53 complexes, ultimately activating *β*‐catenin signaling.^[^ ^16^ ^]^ However, the cross‐talk mechanism between lipid metabolism and Wnt‐*β*‐catenin pathway are still poorly understood.

We describe here a genome‐wide Crispr/Cas9 screen to identify genes that are responsible for imparting resistance to *β*‐catenin inhibitors in Huh7 cells. This identified PMVK as an enzyme that can stabilize *β*‐catenin protein. Mechanistically, PMVK‐generated MVA‐5PP inhibits CKI*α*‐mediated *β*‐catenin phosphorylation at Ser45. This stabilizes the protein by preventing its proteolytic degradation. Additionally, PMVK directly phosphorylates *β*‐catenin at an alternate site, Ser184 to facilitate its nuclear localization. Finally, we describe a novel small molecule inhibitor of PMVK that attenuates *β*‐catenin signaling and potentially represents a novel therapeutics. Collectively, our study reveals a novel, phosphorylation‐dependent mechanism by which the key lipid‐metabolizing enzyme PMVK regulates Wnt‐*β*‐catenin signaling and the ensuing oncogenicity of this otherwise highly regulated pathway.

## 2 Results

### 2.1 Genome‐Wide CRISPR Screen Identifies Regulators of *β*‐Catenin Signaling in Huh7 Cells

**Figure 1:**
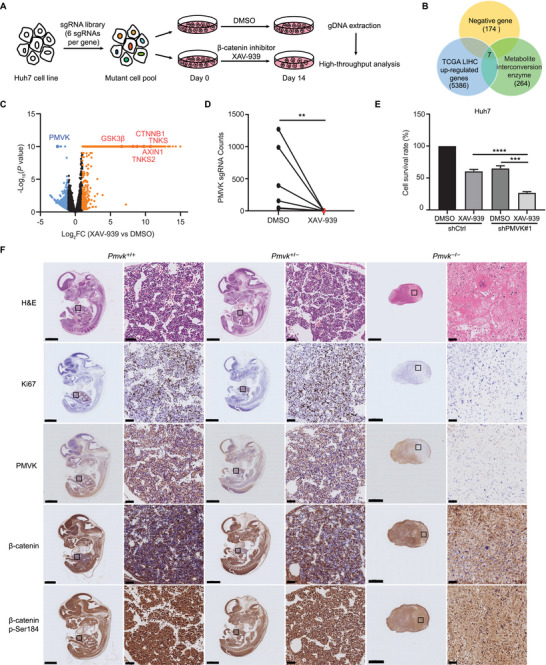
CRISPR‐Cas9 library screens for PMVK stabilizing *β*‐catenin protein levels in Huh7 cells. A) Workflow of GeCKO in Huh7 cells screens to identify potential resistance genes to *β*‐catenin inhibitors. B) Venn diagram showing 174 negative genes (significantly depleted sgRNAs), 264 metabolite interconversion enzymes, and 5386 oncogenes upregulated in HCC. See Table S1, Supporting Information, for details. C) Volcano map showing sgRNA library screening distribution. Blue dots indicate significant deletion of sgRNAs (negative selection). Orange dots indicate significant enrichment of sgRNAs (positive selection). Red dots show screening of positive gene representatives. FC, Fold change. D) Each PMVK sgRNA was analyzed in pairs in the DMSO and XAV‐939 groups. E) Cell viability was determined by MTT after 2 µg shRNA#1 PMVK plasmid transiently transfected with Huh7, DMSO or XAV‐939 (30 *µ* m) treated for 72 h. Data are shown as mean ± SD. F) Representative IHC images of H&E, Ki67, PMVK, total *β*‐catenin and *β*‐catenin p‐Ser184 in wild‐type, PMVK^+/−^ and PMVK^−/−^ E12.5 embryos. Scale bar, 1 mm or 50 µm. The IHC staining experiment was performed twice.

To circumvent the difficulty of targeting the *β*‐catenin signaling pathway itself in HCC, we instead used a human genome‐scale CRISPR‐Cas9 knockout (GeCKO) v2 library to identify genes that support the pathway.^[^ ^17^ ^]^ We chose the CTNNB1 gene wild‐type (WT) HCC cell line Huh7 to avoid the abnormal activation of *β*‐catenin signaling caused by the gene mutations. Preliminary screening showed that the IC_50_ of Huh7 cells for compound XAV‐939, a tankyrase1/2 inhibitor stabilizing of both Axin1 and Axin2, which indirectly promotes the degradation of *β*‐catenin ^[^ ^18^ ^]^ (Figure S1A, Supporting Information). Huh7 cells were then infected with lentiviral stocks encoding the GeCKO v2 library, followed by selection with puromycin. The infected cells were treated with DMSO or XAV‐939 for 14 days, and then analyzed by high‐throughput sequencing for sgRNA enrichment of more than twofold (**Figure** **1**A). Pathway enrichment showed that gRNAs targeted against the Wnt‐*β*‐catenin signaling pathway were significantly enriched as expected (Figure S1B, Supporting Information). Interestingly, protein class analysis showed the highest enrichment to be for “metabolite interconversion enzyme,” implying that these were involved in regulating *β*‐catenin signaling (Figure S1C, Supporting Information). Therefore, we selected 174 significantly down‐regulated enriched genes (potentially stabilizing *β*‐catenin levels, called negative gene) overlapping with metabolite interconversion enzyme, while the screened candidate genes should also be up‐regulated in HCC to fit the role of an oncogene (Figure 1B; Figure S1D and Table S1, Supporting Information). The results showed that PMVK, a metabolic enzyme of the mevalonate pathway, was significantly enriched (Figure 1C,D). However, because the relationship between PMVK and the *β*‐catenin signaling was unclear, PMVK was further investigated as a candidate gene.

### 2.2 Knockout of PMVK Reduces *β*‐Catenin Protein Levels and Blocks Embryonic Development in Mice

Initial experiments showed that XAV‐939 has a stronger inhibitory effect on PMVK knockdown Huh7 cell lines (Figure 1E). To further determine the regulatory role of PMVK on *β*‐catenin, we constructed PMVK knockout mice and found that WT and PMVK^+/−^ mice reproduced normally, but that PMVK homozygous knockout was embryonic lethal (Figure S1E,F, Supporting Information). H&E staining showed no obvious difference in embryonic morphology in PMVK^+/−^ mice compared to WT mice. In addition to PMVK *β*‐catenin protein levels were also significantly reduced in both PMVK^+/−^ and PMVK^−/−^ mice (Figure 1F). Given that Wnt is the major intracellular modality regulating *β*‐catenin signaling and its importance in embryonic development, we analyzed the role of PMVK in Wnt‐*β*‐catenin signaling and our results showed that knockdown of PMVK blocked Wnt3a‐activated *β*‐catenin signaling (Figure S1H, Supporting Information). PMVK knockdown upregulated some *β*‐catenin‐dependent Wnt proteins, which may have triggered a negative feedback (Figure S1I, Supporting Information). These results first suggested that PMVK is required for mouse embryonic development, and that its knockdown PMVK significantly reduces *β*‐catenin protein levels.

### 2.3 PMVK Knockdown Suppresses *β*‐Catenin Signaling Pathway and Tumor Growth

**Figure 2:**
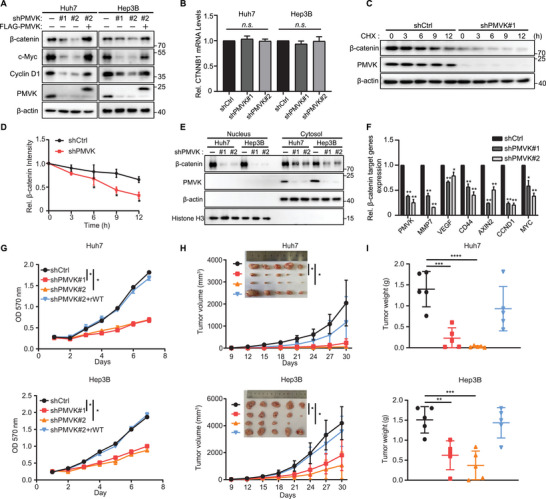
Knockdown of PMVK reduces *β*‐catenin signaling and inhibits tumor growth. A) *β*‐catenin, c‐Myc, Cyclin D1, and PMVK protein expression in the indicated cancer cell lines. B) mRNA expression of *β*‐catenin in the indicated cancer cell lines. C) HEK293 cells were transfected with the indicated vectors. Cells were treated with CHX (50 µg mL^−1^) for the indicated time and the expression of PMVK and *β*‐catenin were analyzed by western blotting. D) The intensity of *β*‐catenin expression for each time point in (C) was quantified by densitometry, with *β*‐actin as a normalizer. E) Distribution of PMVK and *β*‐catenin protein levels in the nucleus or cytoplasm in the indicated PMVK‐knockdown or control cancer cell lines. *β*‐actin as a cytoplasm normalizer and Histone H3 as a nucleus normalizer. F) qRT‐PCR analysis was performed to measure the mRNA levels of PMVK and *β*‐catenin target genes in Huh7 cells. G) Cell proliferation assays were performed in Huh7 (up) and Hep3B (down) cells stably expressing the indicated plasmids. rWT, rescued wild type PMVK. H) Subcutaneous xenograft experiments were performed in Huh7 (up) and Hep3B (down) cells stably expressing the indicated plasmids. *n* = 5 mice per group. I) Subcutaneous xenograft tumor weight for (H). Data are shown as mean ± SD.

To better study how PMVK regulates *β*‐catenin, we generated stable PMVK knockdowns in CTNNB1‐WT Huh7 cells and AXIN1‐mutant Hep3B cells. Each of these dramatically repressed *β*‐catenin protein expression and downstream signaling in a manner that could be rescued by WT PMVK (**Figure** **2**A). The fact that PMVK knockdown did not affect *β*‐catenin mRNA levels indicates that PMVK regulates *β*‐catenin protein levels (Figure 2B). Consistent with this, PMVK knockdown significantly reduced *β*‐catenin protein half‐life while reducing its nuclear localization (Figure 2C–E) and the expression of known *β*‐catenin target genes (Figure 2F). Conversely, overexpression of PMVK retarded *β*‐catenin degradation while promoting the nuclear localization (Figure S2A–C, Supporting Information). Consistent with this and the accompanying reduction in c‐Myc and Cyclin D1 levels, PMVK knockdown significantly inhibited cell proliferation in a manner that could be rescued by PMVK re‐expression (Figure 2G). Similar results were obtained in a mouse xenograft tumor model (Figure 2H,I and Figure S2D, Supporting Information). Thus, PMVK appears to stabilize the *β*‐catenin protein, which is in turn necessary to maintain cell proliferation and tumor growth.

It has been shown that prenylation‐modified Rho‐GTPase can activate YAP/TAZ signaling, which contributes to *β*‐catenin activity.^[^ ^19^ ^]^ To investigate whether PMVK stabilizes *β*‐catenin protein levels in relation to prenylation modification, we used shRNAs knockdown KRas protein. Results showed that knockdown of KRas did not affect *β*‐catenin levels (Figure S2E, Supporting Information). To investigate whether PMVK‐mediated regulation of *β*‐catenin is related to cholesterol levels, we supplemented PMVK‐expressing HCC cells with cholesterol and showed that this only partially restored cell proliferation (Figure S2F, Supporting Information). This suggests that cholesterol may be involved in the regulation of *β*‐catenin by PMVK but is not the main cause. Combined with the results of previous experiments in which knockdown of PMVK blocked Wnt3a‐activated *β*‐catenin (Figure S1H, Supporting Information), we hypothesized that PMVK may directly interact with and stabilize *β*‐catenin.

### 2.4 PMVK‐Mediated Phosphorylation of *β*‐Catenin at Ser184 Enhances Its Stability and Promotes Its Nuclear Translocation

**Figure 3:**
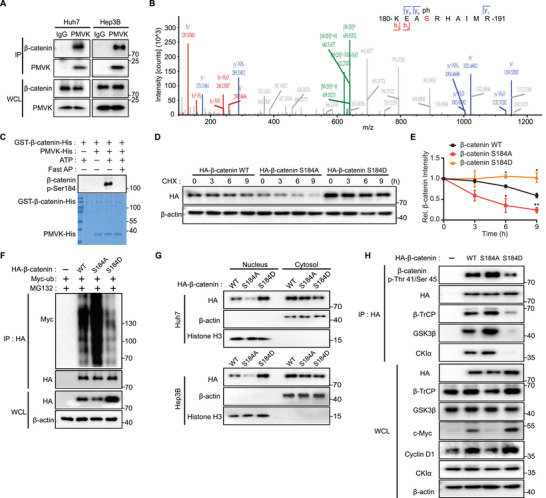
PMVK‐mediated *β*‐catenin Ser184 phosphorylation, protein stability and nuclear localization. A) Endogenous interaction between PMVK and *β*‐catenin in Huh7 and Hep3B cell lines. Anti‐PMVK Antibody (H‐9) from mouse as an IP antibody. Mouse lgG was used as a negative control. WCL, Whole cell lysate. B) Tandem mass spectrometry spectrum of *β*‐catenin pSer184. Detected productions are indicated in red (b ions) and blue (y ions). See Table S2, Supporting Information, for details. C) Recombinant purified GST‐*β*‐catenin‐His and PMVK‐His proteins were subjected to an in vitro kinase reaction. *β*‐catenin phosphorylation was identified with *β*‐catenin p‐Ser184 antibody. Fast AP, Fast alkaline phosphatase. D) HEK293 cells were transfected with the indicated vectors. Cells were treated with CHX (50 µg mL^−1^) for the indicated times and the levels of different *β*‐catenin mutants were analyzed by Western blotting. E) The expression intensity of different *β*‐catenin mutants at each time point in (D) was quantified by densitometry, with *β*‐actin as a normalizer. Data are shown as mean ± SD. F) HEK293 cells were transfected with the indicated plasmids, treated with MG132 for 6 h and then lysed in RIPA buffer. Immuno‐precipitated HA‐*β*‐catenin proteins were subjected to immunoblot assay with Myc‐tag antibody. G) Distribution of different *β*‐catenin mutants protein levels in the nucleus or cytoplasm in Huh7 and Hep3B cells transfected with the indicated plasmids. *β*‐actin as a cytoplasm normalizer and Histone H3 as a nucleus normalizer. H) HEK293 cells were transfected with the indicated plasmids and then lysed in RIPA buffer. Immuno‐precipitation of different *β*‐catenin mutants proteins with anti‐HA magnetic beads and subjected to immunoblot assay.

Co‐immunoprecipitation (co‐IP) experiments with the endogenous proteins indicated that PMVK interacts directly with *β*‐catenin (**Figure** **3**A). Subsequent domain mapping demonstrated that this association requires PMVK's kinase domain and *β*‐catenin's N‐terminal domain (Figure S3A,B, Supporting Information). Given that PMVK is a metabolic kinase,^[^ ^21^ ^]^ we asked whether it can directly phosphorylate *β*‐catenin by performing phosphorylation experiments in vitro and identifying phosphorylation sites by mass spectrometry (Figure 3B and Table S2, Supporting Information). These studies showed that the evolutionarily conserved *β*‐catenin Ser184 residue was directly phosphorylated by PMVK and this was confirmed using a pSer184‐specific antibody (Figure 3C and Figure S3C, Supporting Information).

We then asked whether PMVK‐mediated *β*‐catenin phosphorylation was responsible for stabilizing *β*‐catenin. To this end, we generated S184A (phosphorylation “dead”) and S184D (phosphomimetic) phosphorylation site mutants and determined their half‐lives following cycloheximide (CHX) block. The S184A mutant protein showed a significant decrease in its half‐life while S184D’s half‐life was prolonged (Figure 3D,E). Consistently, S184A showed increased levels of ubiquitination while S184D showed the opposite (Figure 3F). Furthermore, *β*‐catenin S184A showed markedly reduced nuclear localization (Figure 3G). In addition, immunofluorescence experiments also showed that S184A was mainly localized in the cytoplasm while S184D was significantly localized in the nucleus compared to *β*‐catenin WT. This further suggested that PMVK‐mediated phosphorylation of *β*‐catenin at Ser184 promotes its nuclear localization (Figure S3E, Supporting Information). IHC staining of mouse embryos also showed that endogenous phosphorylated *β*‐catenin Ser184 preferentially localizes to the nucleus (Figure 1E).

In adult hepatocytes, CKI*α* normally phosphorylates *β*‐catenin S45, which initiates GSK3*β*‐mediated *β*‐catenin phosphorylation as well as *β*‐TrCP‐mediated *β*‐catenin ubiquitination and proteasome‐mediated degradation.^[^ ^20^ ^]^ Therefore, we asked whether PMVK‐mediated *β*‐catenin Ser184 phosphorylation is regulated via changes in the above degradation complex proteins. The results showed that, whereas knockdown and over‐expression of PMVK respectively decreased and increased *β*‐catenin p‐Ser184 phosphorylation, neither affected the levels of CKI*α*, GSK3*β*, and *β*‐TrCP in HCC cells (Figure S3D, Supporting Information). However, *β*‐catenin S184D showed significantly reduced association with CKI*α*, GSK3*β*, and *β*‐TrCP compared with S184A, whereas the phosphorylation of both Thr41 and Ser45 was significantly increased in S184A. Overexpression of WT *β*‐catenin or S184D, but not S184A, also activated *β*‐catenin downstream signaling (Figure 3H). Together the above results indicated that PMVK promotes *β*‐catenin protein stability and nuclear translocation by phosphorylating its Ser184 site, which in turn interferes with the de‐stabilizing phosphorylation events involving *β*‐catenin residues 41 and 45.

### 2.5 PMVK‐Generated MVA‐5PP Competitively Binds CKI*α* Causing Stabilization of *β*‐Catenin

**Figure 4:**
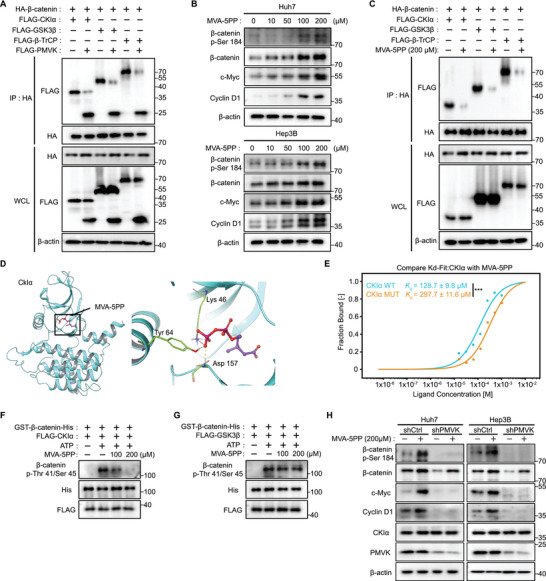
PMVK‐produced MVA‐5PP competitively binds CKI*α* to stabilize *β*‐catenin. A) HEK293 cells were transfected with the indicated plasmids and then lysed in RIPA buffer. Immuno‐precipitated HA‐*β*‐catenin proteins were subjected to immunoblot assay. B) MVA‐5PP was added to Huh7 and Hep3B cells at the indicated final concentrations and subjected to immunoblot assay *β*‐catenin (total and p‐S184 form), c‐Myc and Cyclin D1 protein levels. C) HEK293 cells were transfected with the indicated plasmids, supplemented with MVA‐5PP (200 *µ* m) and then lysed in RIPA buffer. Immuno‐precipitation of HA‐*β*‐catenin proteins were subjected to immunoblot assay. D) The molecular docking of MVA‐5PP and CKI*α* is shown based on the crystal structure of CKI*α* (PDB code: 6GZD). Lys 46, Tyr 64, and Asp 157 indicate the bound residues. E) *K*d values were determined by MST for MVA‐5PP bound to purified human CKI*α* or CKI*α* mutant (K46A, Y64A and D157A) proteins. Data are shown as mean ± SD. F) Purified recombinant GST‐*β*‐catenin‐His protein and FLAG‐CKI*α* protein were subjected to in vitro phosphorylation assays at different concentrations of MVA‐5PP. Immunoblotting was performed using *β*‐catenin p‐Thr41/Ser45 antibody. G) Recombinant GST‐*β*‐catenin‐His and FLAG‐GSK3*β* proteins were subjected to in vitro phosphorylation assays at different concentrations of MVA‐5PP followed by immunoblotting using *β*‐catenin p‐Thr41/Ser45. H) Immunoblot assay of *β*‐catenin (total and p‐S184 form), c‐Myc, Cyclin D1, and CKI*α* protein levels after MVA‐5PP (200 *µ* m) treatment in the indicated PMVK‐knockdown or control cancer cell lines.

*β*‐catenin's stability and turnover are regulated by the destruction complex.^[^ ^21^ ^]^ Our new findings show that, by directly phosphorylating *β*‐catenin, PMVK participates directly in this regulation. We therefore next asked whether the interaction between PMVK and *β*‐catenin occurs within the destruction complex or elsewhere. Co‐IP results showed that ectopic PMVK expression reduced the interaction between *β*‐catenin and several destruction complex‐associated proteins (**Figure** **4**A). Interestingly, the over‐expression of a metabolically inactive mutant of PMVK, PMVK‐(17‐23),^[^ ^22^ ^]^ did not affect the interaction of *β*‐catenin with the destruction complex whereas PMVK WT did (Figure S4A, Supporting Information). This result suggested that the PMVK‐mediated stabilization of *β*‐catenin protein levels relies upon downstream metabolites. Indeed, addition of the known PMVK metabolite MVA‐5PP to Huh7 and Hep3B cells increased their levels of both total and p‐S184 *β*‐catenin and the down‐stream *β*‐catenin targets c‐Myc and Cyclin D1 in a concentration‐dependent manner (Figure 4B). Moreover, the addition of MVA‐5PP to cells lacking PMVK reduced *β*‐catenin's association with the destruction complex proteins (Figure 4C). Reverse in silico screening of the destruction complex's structure indicated a potential association between MVA‐5PP and CKI*α* (8.2 kcal mol^−1^) (Figure S4B, Supporting Information).

Diphosphomevalonate decarboxylase (MVD) catalyzes the fourth step of mevalonate biosynthesis involving the cation‐dependent reaction of MVA‐5PP with ATP to form isopentenyl diphosphate and ADP to release a carbon dioxide and a phosphate group, which binds to MVA‐5PP as a positive control (8.6 kcal mol^−1^) (Figure S4B, Supporting Information).^[^ ^23^ ^]^ To verify this result, we used Microscale thermophoresis (MST) to confirm binding of MVA‐5PP to the K46, Y64, and D157 sites of CKI*α*, with much less binding being seen with a mutant CKI*α* (K46A, Y64A, and D157A) (Figure 4D,E). In vitro phosphorylation assays also showed that MVA‐5PP inhibited, in a concentration‐dependent manner, the phosphorylation of *β*‐catenin S45 by CKI*α* but not *β*‐catenin T41 by GSK3*β* (Figure 4F,G). Finally, shRNA‐mediated depletion of MVD, which served an independent means of increasing MVA‐5PP levels, likewise increased *β*‐catenin protein. This suggested that the stabilization of *β*‐catenin was primarily caused by MVA‐5PP rather than its downstream metabolites (Figure S4C, Supporting Information). In addition, the supplementation of cells with exogenous MVA‐5P increased the levels of total and p‐S184 *β*‐catenin as well as c‐Myc and Cyclin D1, indicating that PMVK's upstream metabolites also contribute to the activation of *β*‐catenin signaling, but that the primary effector is MVA‐5PP (Figure S4D, Supporting Information).

Cholesterol deficiency stimulates that transcription factor SREBP2, which in turn increases the expression of genes involved in the cholesterol biosynthesis pathway.^[^ ^24^ ^]^ To evaluate whether this can promote *β*‐catenin p‐Ser184 and *β*‐catenin signaling, we treated HCC cells with methyl‐*β*‐cyclodextrin (M‐*β*‐CD) which depletes cholesterol.^[^ ^25^ ^]^ Subsequent immunoblotting showed that M‐*β*‐CD treatment partially decreased the levels total and p‐S184 *β*‐catenin. This suggested that *β*‐catenin S184 phosphorylation is dependent on certain minimal levels of MVA‐5PP (Figure S4E, Supporting Information). The data also suggest that the product of PMVK, namely MVA‐5PP, rather than PMVK itself, competitively binds to CKI*α* to prevent the phosphorylation‐dependent degradation of *β*‐catenin.

Finally, in vitro supplementation with MVA‐5PP restored *β*‐catenin levels in PMVK knockdown cells but failed to rescue *β*‐catenin p‐Ser184 or the downstream signaling defects. This may have been due to the lack of PMVK‐mediated *β*‐catenin Ser184 phosphorylation (Figure 4H).

### 2.6 PMVK Knockout in Hepatocytes Blocks HCC Generation in Mice

**Figure 5:**
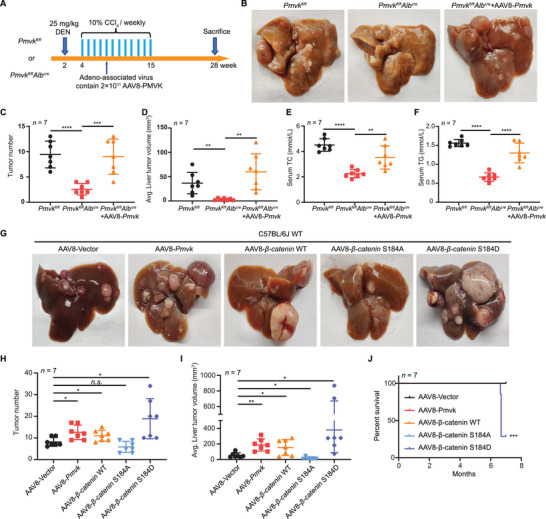
Liver‐specific deletion of PMVK inhibits HCC growth in mice. A) Schematic overview of DEN/CCl_4_‐induced HCC mice model. During this period, adeno‐associated virus containing PMVK were given via tail‐vein injection. B) Liver images extracted from the indicated mice. C) Tumor number of each liver from (B). D) Average tumor volume of each liver from (B). E) Serum TC levels in mice from (B). F) Serum TG levels in mice from (B). G) Representative IHC images of H&E and Ki67 in tumor tissues from (B). H) Adeno‐associated virus containing the indicated plasmids were delivered via tail‐vein injection. Liver images extracted from the indicated mice. I) Tumor number of each liver for (H). J) Average tumor volume of each liver for (H). K) Percent survival of mice for (H).

To determine the role of PMVK in HCC development in vivo, PMVK hepatocyte‐specific conditional knockout (CKO) mice were constructed (Figure S5A, Supporting Information). Immunoblotting confirmed that these mice also had significantly reduced levels of both total and p‐S184 *β*‐catenin (Figure S5B, Supporting Information). Although the results of Ki67 staining showed that cell proliferation was suppressed in PMVK *^flfl^* Alb*^cre^* mice (Figure S5C, Supporting Information), there were no significant differences in body or liver weights compared to WT mice at 8 weeks of age (Figure S5D,E, Supporting Information). To evaluate PMVK's role on HCC development, we generated tumors in both groups of mice with DEN/CCl_4_ (**Figure** **5**A). CKO mice had both significantly reduced tumor numbers and smaller tumor volumes compared to control WT mice (Figure 5B–D). Serum TC and TG levels were also significantly reduced in the CKO group (Figure 5E,F) and Ki‐67 staining showed that tumor proliferation was significantly inhibited (Figure S5F, Supporting Information). Adeno‐associated virus 8 (AAV8) restoration of PMVK expression promoted tumor growth and increased serum levels of TC and TG in CKO mice (Figure 5B–F). To further investigate the effects of PMVK and *β*‐catenin Ser184 phosphorylation on tumor growth, we delivered PMVK or *β*‐catenin (WT and S184A/D) via AAV8 in WT mice. Our results showed that overexpression of PMVK, *β*‐catenin WT or S184D significantly promoted HCC growth (Figure 5G–I). S184D also significantly reduced the survival rate (Figure 5J). Collectively, these data suggest that liver‐specific PMVK deletion significantly slows HCC development by reducing cell proliferation and cholesterol and triglyceride levels whereas PMVK‐mediated phosphorylation of *β*‐catenin Ser184 strongly promotes tumor growth.

### 2.7 PMVK Is Overexpressed in HCC Patients and Correlates with Poor Prognosis

**Figure 6:**
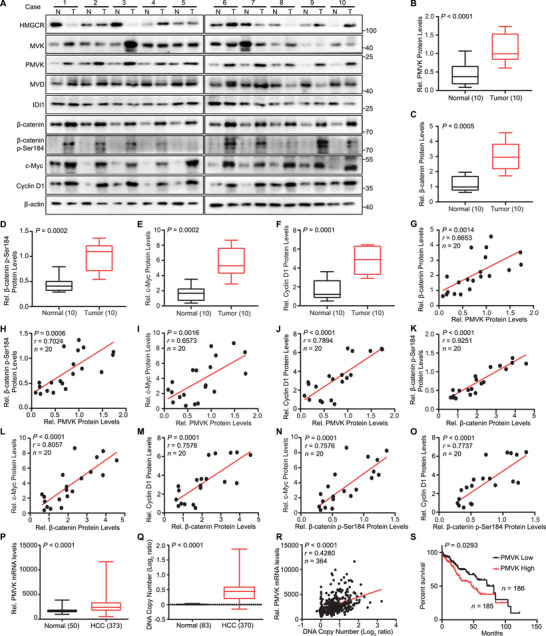
PMVK expression is increased in human HCC and correlates with poor survival. A) Western blot analysis for the indicated proteins in human HCCs. T, tumor; N, adjacent normal tissue. B–F) The intensities of PMVK, *β*‐catenin (total and p‐S184), c‐Myc and Cyclin D1 for (A) were quantified by densitometry, with *β*‐actin as a normalizer. Data are shown as mean ± SD. G–O) Correlation of different protein levels in HCC tissues from (A). Each point is an individual sample. P) PMVK mRNA levels in normal liver and HCCs from TCGA. Q) PMVK gene copy number in normal liver and tumor tissues from TCGA. R) Correlation of PMVK mRNA level with its DNA copy number in HCC tissues from TCGA dataset. Each point is an individual sample. S) Kaplan–Meier curves with univariate analysis of the survival of patients with HCC based on high versus low expression of PMVK. Data are shown as mean ± SD. The *P* values were determined by paired two‐sided Student's *t*‐test for (B–F) and unpaired two‐sided Student's *t*‐test for (P) and (Q). The correlation coefficient (*r*) and *p* values in (G–O and R) were determined using two‐tailed Pearson correlation analysis.

In tumor samples from 10 HCC patients, PMVK, *β*‐catenin (total and p‐S184 form), c‐Myc and Cyclin D1 were significantly upregulated relative to matched normal liver (**Figure** **6**A–F). In addition, PMVK positivity correlated with *β*‐catenin protein levels (Figure 6G), in contrast to other enzymes of the mevalonate pathway (Figure S6A–D, Supporting Information). PMVK also positively correlated with *β*‐catenin p‐Ser184, which in turn correlated with the levels of downstream target gene proteins (Figure 6H–O). PMVK was also highly expressed in HCCs from the TCGA database (Figure 6P). Similar findings were shown in the GEO databases (GSE45436, GSE25097, GSE102083, and GSE14520). PMVK mRNA levels also positively correlated with the expression of *β*‐catenin target genes in the TCGA‐LIHC and GSE14520 dataset (Figure S6E–K, Supporting Information). The PMVK gene also tended to be highly amplified in HCC samples, thus explaining the basis for its over‐expression (Figure 6Q,R). Finally, PMVK upregulation correlated with poor HCC patient survival and advanced clinical stage (Figure 6S and Figure S6L, Supporting Information). Together, these data identify PMVK as a potential therapeutic target in HCC.

### 2.8 A Small Molecular Inhibitor of PMVK, Reduces HCC Growth In Vivo

**Figure 7:**
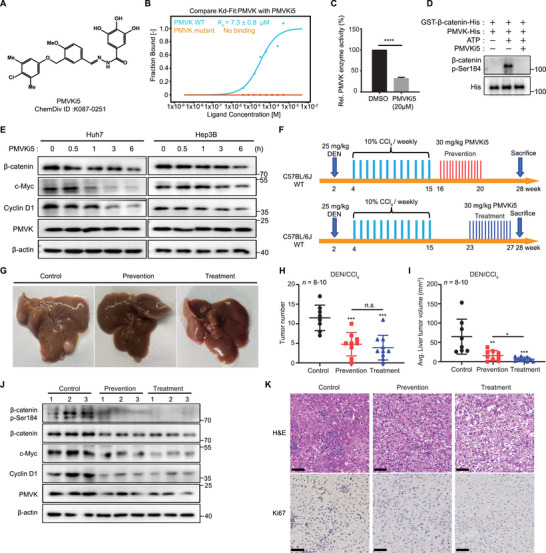
The PMVK inhibitor, PMVKi5, suppresses HCC growth in vitro and in vivo. A) Chemical structure formula of PMVKi5. B) *K*d values were determined as the binding of PMVKi5 to purified human PMVK or PMVK mutant (K17A, S20A, G21A, K22A, and D23A) proteins using MST. C) In vitro kinase activity assays D) Reco9mnbinant GST‐*β*‐catenin‐His and PMVK‐His were used for in vitro kinase reactions, with or without the addition of 20 *µ* m PMVKi5. *β*‐catenin phosphorylation was identified with *β*‐catenin p‐Ser184 antibody. E) Immunoblot for the indicated proteins in Huh7 and Hep3B cell lines following exposure to 20 *µ* m PMVKi5 for 6 h. F) Schematic overview of DEN/CCl4‐induced HCC mice model. For the prevention group, mice were treated between 16 and 20 weeks, with 30 mg kg^−1^ PMVKi5 i./p. every 2 days for a total of 14 doses for. In the treatment group, mice were treated from 23 to 27 weeks. After 28 weeks, livers were extracted. G) Liver images from the indicated mice. H) Tumor number of each liver for (G). I) Average tumor volume of each liver for (G). J) Immunoblot analysis for the indicated proteins in tumor tissues from (G). K) Representative IHC images of H&E and Ki67 in tumor tissues from (G). Scale bar, 50 µm.

Using a virtual screen, we identified the putative PMVK inhibitor top 5 (PMVKi5) (C_24_H_23_ClN_2_O_6_,N‐[(E)‐[3‐[(4‐chloro‐3,5‐dimethylphenoxy)methyl]‐4‐methoxyphenyl]methylideneamino]‐3,4,5‐trihydroxybenzamide) from a library of 1.7 million ChemDiv small molecule compounds (**Figure** **7**A). The molecular docking study based on the crystal structure PMVK showed that PMVKi5 contacts PMVK residues Lys17, Ser20, Gly21, Lys22, Asp23, Arg73, and Arg141 (Figure S7A, Supporting Information). In support of this model, the results of MST showed PMVKi5 to have a higher affinity for WT PMVK than for mutants harboring any of the above‐discussed residues (Figure 7B). In vitro studies showed PMVKi5 to have IC_50_’s of 7.05 and 18.74 *µ* m in Huh7 and Hep3B cell lines, respectively (Figure S7B, Supporting Information). Given that residues 17–23 of PMVK are necessary for ATP‐binding,^[^ ^22^ ^]^ we speculated that PMVKi5 acts as an ATP mimetic. Indeed, an in vitro kinase assays showed that PMVKi5 significantly inhibited ATP consumption by PMVK and its phosphorylation of *β*‐catenin (Figure 7C,D). Finally treatment of Huh7 and Hep3B cells with 20 *µ* m PMVKi5 for 6 h significantly inhibited *β*‐catenin signaling without altering PMVK protein levels (Figure 7E).

To further assess the role of PMVKi5 in tumorigenesis, we again utilized the DEN/CCl_4_ HCC model in which mice were treated with PMVKi5 (30 mg kg^−1^ intraperitoneally) every 2 days for a total of 14 doses (Figure 7F). Both prevention and treatment group mice showed significant reduced tumor burdens compared to control tumor‐bearing mice (Figure 7G–I). PMVKi5 treatment also inhibited *β*‐catenin protein levels and downstream targets in mouse tumors (Figure 7J). Ki67 staining confirmed that PMVKi5 significantly inhibited tumor proliferation (Figure 7K).

To further evaluate PMVKi5 efficacy in other cancers, we used the AOM/DSS induced colorectal cancer (CRC) mouse model (Figure S7G, Supporting Information). Similarly, PMVKi5 also inhibited tumorigenesis in this model (Figure S7H–K, Supporting Information). Collectively, these data confirm our previous results by showing that PMVKi5 blocks *β*‐catenin signaling by inhibiting PMVK.

Importantly, PMVKi5 did not alter mouse body weight or serum ALS, ATL levels, indicating that PMVKi5 treatment does not cause liver injury (Figure S7D–F,K, Supporting Information). PMVKi5 thus represents a promising lead compound that should be safe and effective for the prevention and treatment of HCC and/or CRC.

## 3 Discussion 

The WNT/*β*‐catenin signaling pathway is often activated in cancer due to the aberrant accumulation and nuclear localization of its effector *β*‐catenin.^[^ ^10^ ^]^ Although many *β*‐catenin inhibitors have been screened in the past decades, the eventual development of resistance has tended to stymie such efforts. There is thus an urgent need to screen for new targets that regulate *β*‐catenin indirectly.^[^ ^26^ ^]^ This consideration was the primary motivation for our genome‐wide Crispr screen that identified PMVK as one such potential target.

Of note, metabolic enzymes were significantly enriched in the above‐mentioned screen. Although metabolic reprogramming is one of the hallmarks of malignancy, its relationship to *β*‐catenin signaling has, until now, been unclear.^[^ ^27^ ^]^ Our Crispr/Cas9 screen led us to focus our attention on the mevalonate pathway enzyme PMVK as a novel target that regulates *β*‐catenin signaling. As a result, we have found that PMVK plays key roles in embryonic development and tumor growth, while PMVK‐mediated *β*‐catenin p‐Ser184 phosphorylation is also significantly upregulated in both key nodes (Figure S5G, Supporting Information). The importance of this activity may be due to the uniqueness of the mevalonate pathway as the upstream pathway of cholesterol synthesis and the importance of *β*‐catenin signaling in embryonic development and malignancy.^[^ ^28^ ^]^

Previous studies have also shown that the mevalonate metabolic pathway contributes prominently to prominent tumor growth, including the ability of MVA‐5P to stabilize mutant p53.^[^ ^7^ ^]^ In addition, mevalonate pathway metabolites are also important for prenylation, geranylgeranylation, and farnesylation of several oncogenic proteins, such as Rho, Rac, Rab, and Ras.^[^ ^29^ ^]^ Although geranylgeranylation can activate the YAP/TAZ signaling pathway and thus promote the *β*‐catenin signaling pathway,^[^ ^19^, ^30^ ^]^ our results show that this activity is not involved in the PMVK‐mediated regulation of *β*‐catenin signaling (Figure S2E, Supporting Information). In addition, knockdown of PMVK resulted in blocked synthesis of the mevalonate pathway, which also impaired cholesterol levels. Previous studies have shown that accumulated cholesterol stabilizes *β*‐catenin signaling.^[^ ^31^ ^]^ But in our study we showed that supplementation of additional cholesterol levels in HCC cells with knockdown PMVK only partially restored cell proliferation levels, suggesting that although cholesterol is an important factor for cell proliferation, it is not a major factor in the regulation of *β*‐catenin signaling by PMVK (Figure S2F, Supporting Information).

Interestingly, PMVK knockdown also impaired Wnt3a‐activated *β*‐catenin signaling, suggesting that the mechanism of PMVK involves the disruption complex or the nuclear localization of *β*‐catenin. qRT‐PCR showed that PMVK knockdown increased some Wnt family transcript levels, possibly due to a classical feedback mechanism triggered by the loss of *β*‐catenin signaling. This prompted us to explore whether PMVK interacts directly with *β*‐catenin. As a result, we determined that PMVK directly phosphorylates *β*‐catenin Ser184, thereby increasing *β*‐catenin protein stability, promoting its nuclear translocation and increasing target gene expression. Most HCCs contain mutations in *CTNNB1*, which stabilize *β*‐catenin by preventing its association with the destruction complex. However, we found that knockdown of PMVK still destabilized *β*‐catenin and impaired its downstream signaling in Hep3B (AXIN1 mutant) cells, in which the destruction complex is non‐functional. This suggested that, by phosphorylating the critical Ser184 residue, PMVK activates *β*‐catenin via a destruction complex‐independent mechanism. In the HCC mouse model, overexpression of *β*‐catenin p‐Ser184 strongly promoted tumor growth. Although stabilizing *β*‐catenin indirectly promotes its nuclear localization, the actual mechanism by which *β*‐catenin Ser184 phosphorylation accomplishes this requires further clarification. Regardless of the actual means by which this is accomplished, *β*‐catenin Ser184 phosphorylation serves as a new and novel marker for *β*‐catenin pathway activation.

PMVK phosphorylation of *β*‐catenin Ser184 is dependent on its classical metabolic enzyme function. Because PMVK mutants cannot affect the interaction of *β*‐catenin with the destruction complex, this implies that the downstream metabolites of PMVK may cooperate with mutations in the destruction complex or in *β*‐catenin itself to further stabilize the protein, promote its nuclear localization and enhance it's signaling. Interestingly, the PMVK metabolite, MVA‐5PP similarly inhibited the interaction of *β*‐catenin with the destruction complex with similar consequences. We found that the mechanistic underpinnings of this activity involves the binding of MVA‐5PP to CKI*α*. Genetic knockdown of MVD also increased *β*‐catenin protein level. This suggests that PMVK stabilization of *β*‐catenin is dependent on its metabolite MVA‐5PP rather than on the mevalonate pathway metabolite isopentenyl diphosphate or other intermediates. In the developing chicken liver, MVK and PMVK remain highly active for the first 5 days, while the activity of MVD is low.^[^ ^32^ ^]^ This suggests that increased MVA‐5PP may be elevated in a way that satisfies the strong dependency on *β*‐catenin signaling during early development.^[^ ^33^ ^]^ Collectively, we uncovered a non‐canonical function of MVA‐5PP by which it and the PMVK enzyme that generates it, play synergistic roles in regulating the *β*‐catenin signaling pathway.

Analysis of clinical samples and data from TCGA indicated that PMVK is highly expressed in HCC, likely due to its genomic amplification. PMVK levels were also found to correlate closely with clinical stage and *β*‐catenin p‐Ser184 correlated with total *β*‐catenin and its downstream signals. This indicates that PMVK and *β*‐catenin p‐Ser184 protein levels are potentially useful as prognostic markers in HCC. Finally PMVK inhibitors represent a novel targeted therapy directed against a previously unsuspected target that drives HCC progression and may be useful in CRC as well. We also evaluated other inhibitors of the mevalonate pathway on PMVK‐regulated *β*‐catenin signaling. Simvastatin showed the same effect as PMVKi5 on *β*‐catenin, due to the fact that statins block the mevalonate pathway at its source (also blocking the synthesis of MVA‐5PP). Zoledronic acid instead promotes *β*‐catenin protein levels, the target of zoledronic acid is the inhibition of farnesyl pyrophosphate synthesis which may indirectly contribute to the accumulation of upstream metabolites, similar to knockdown of MVD (Figure S7C, Supporting Information).

In conclusion, we have demonstrated how the mevalonate pathway enzyme PMVK controls *β*‐catenin signaling. In doing so, we have revealed a new mechanism by which metabolic reprogramming controls HCC and CRC pathogenesis. PMVK inhibition, perhaps with PMVKi5 or its analogs thus represents a promising and rational therapeutic strategy.

## 4 Experimental Section 

### Vectors, Reagents, Antibodies, Cell Culture, and Stable Cell Lines

Human PMVK shRNAs (Table S3, Supporting Information) were expressed in the PLKO.1 vector. The open reading frames of human PMVK, *β*‐catenin, and CKI*α* were amplified and cloned into pHAGE‐CMV‐MCS‐PGK‐3×FLAG and pCMV‐HA vectors. Mutations in the PMVK, *β*‐catenin, and CKI*α* cDNA sequences were generated by overlap extension PCR. Primers for constructs used in this study are listed in Table S4, Supporting Information, and were synthesized by GeneCreate Biological Engineering Co. Ltd (Wuhan, China). Transfection and the establishment of stable cell lines were performed as previously described.^[^ ^34^ ^]^

5‐diphosphomevalonate was purchased from Sigma‐Aldrich. More reagent information in this study is listed in Table S5, Supporting Information. Anti‐HA magnetic beads and anti‐FLAG affinity agarose beads were purchased from Selleck (Houston, USA). Antibodies used in this study are listed in Table S6, Supporting Information.

Human HEK293 cells were obtained from the American Type Culture Collection in 2009. Human liver tumor‐derived cell lines Huh7 and Hep3B were obtained from the China Center for Type Culture Collection and the Cell Bank of the Type Culture Collection of The Chinese Academy Sciences and cultured as previously described.^[^ ^34^ ^]^ All cells were regularly authenticated by short tandem repeat analyses, were tested for the absence of Mycoplasma and were used within five passages after thawing.

### Animal Experiments

All animal studies were approved by the Animal Care Committee of Wuhan University (No. WQ20210130). 4‐week‐old female BALB/c nude mice were purchased from Gempharmatech (Jiangsu, China). PMVK*^−/−^* mice were generated, bred and raised at Cyagen Biosciences with CRISPR‐Cas9 technology to delete exon E1‐E5 of the PMVK gene. PMVK*^fl/fl^* mice (Cyagen Biosciences) have loxP sites flanking exons 2 and 5 of the PMVK gene. Alb‐Cre mice were kindly provided by Prof. Yong Liu (Wuhan University). To generate PMVK*^fl/fl^* Alb*^cre^* mice, PMVK*^fl/fl^* mice were crossed with Alb‐Cre mice. All mouse genotype identification primer sequences used are shown in Table S7, Supporting Information.

For xenograft experiments, tumor growth was monitored every 3 days for a total period of 30 days. Tumor volumes were calculated by the equation *V* (mm^3^) = *a* × *b* × (*a* + *b*)/2, where *a* is the length and *b* is the width.

For induction of HCCs, C57BL/6J, PMVK*^fl/fl^* and PMVK*^fl/fl^* Alb*^cre^* male mice were intraperitoneally administrated 25 mg kg^−1^ of *N*‐Nitrosodiethylamine (DEN) (Sigma) at 2 weeks after birth. From 4 to 15 weeks, mice were intraperitoneally injected with 10% CCl_4_ (5 mL kg^−1^) weekly. After 28 weeks, livers were examined. For induction of CRCs, C57BL/6J mice were intraperitoneally administrated AOM (12.5 mg kg^−1^) at 8 weeks after birth. From 9, 12, and 15 weeks, 2.5% DSS was added to the drinking water of mice for 1 week, after 25 weeks, the colons were examined.

Littermate mice were randomly divided into control and various treatment groups including: control, PMVKi5 prevention, and PMVKi5 treatment. All cohorts were maintained as blinded cohorts following these treatments.

### Human Genome‐Wide CRISPR Screening

Amplification of the sgRNA library followed the description of Feng Zhang et al.^[^ ^17^, ^35^ ^]^ Twelve T225 culture flasks were prepared for library virus generation according to the HEK293 culture conditions described above. Each flask was transfected with 20 µg of library plasmid (10 µg each of libraries A and B), 10 µg of pMD2.G and 15 µg of psPAX2 were transfected using 100 *µ*L of Lipofectamine 2000 and 200 *µ*L of Plus Reagent. The medium was replaced after 24 h and the cell supernatants were collected at 48 and 72 h of transfection and passed through 0.45 µm filters. The packed lentiviral library was then used to infect at least 5 × 10^7^ Huh7 cells in the presence of 1 µg mL^−1^ polybrene, ensuring a multiplicity of infection (MOI) of 0.3 so that most cells receive only one stably integrated RNA guide. 24 h after infection, puromycin was added to select for stably transduced cells. Allow the cell count to expand to >8 × 10^7^, ensuring that the initial sieve cell count is a library coverage multiple >300×. Each group of 4 × 10^7^ cells were treated with vector and XAV393 (30 *µ* m), respectively, and cells were passaged every 2 days for a total of 14 days of drug treatment. All cells were collected and genomic DNA was extracted, enriched for fragments containing sgRNA sequences using amplification primers (forward primer, ATGGACTATCATATGCTTACCGTA; reverse primer, AGTACACGACATCACTTTCCC) and sequenced on an Illumina NovaSeq 6000 following a second round of PCR reactions with the addition of splice primers and indexT sequences used to identify different samples. The number of sgRNAs of XAV‐939 and vehicle was identified using the MAGeCK algorithm and analyzed using DESEq2 to obtain significantly enriched or depleted sgRNAs.

### Immunohistochemistry and Immunofluorescence

Liver sections or mouse embryos were fixed in 10% formalin and embedded in paraffin according to standard protocols. Liver sections or mouse embryo were sectioned and stained with H&E, Ki67, PMVK, *β*‐catenin, and *β*‐catenin p‐Ser184. Images were obtained on a Leica Aperio VERSA 8 microscope.

Immunofluorescence analysis was performed as previously described.^[^ ^34^ ^]^ Briefly, HEK293 cells were transfected with the indicated vectors. 48 h later, they were fixed in 4% paraformaldehyde for 15 min, followed by 0.2% Triton X‐100 permeabilization for 5 min and 3% BSA blocking for 1 h. After incubation with primary antibody against FLAG (1:200) overnight in 4 °C, the cells were washed well with PBST and incubated with ABflo 488‐conjugated goat anti‐rabbit (1:500) for 1 h at room temperature. After additional extensive washing, the cells were labeled with DAPI (5 µg mL^−1^) for 5 min at room temperature. Fluorescence imaging was performed with a LSM800 confocal laser scanning microscope.

### Co‐IP and Immunoblotting

Transfected cells were lysed in 1 mL lysis buffer [50 mm Tris (pH 7.4), 150 mm NaCl, 1% NP‐40, 0.5% Sodium deoxycholate, 0.1% SDS, 5 mm EDTA, 10 mg mL^−1^ aprotinin, 10 mg mL^−1^ leupeptin, and 1 mm PMSF]. Sepharose beads were washed three times with 1 mL lysis buffer containing 0.1% NP‐40. Co‐IP and immunoblot analysis were performed as described.^[^ ^36^ ^]^

### qRT‐PCR

Total RNA was isolated using Trizol followed by DNase treatment. Reverse transcription was performed with a cDNA Synthesis Kit and qPCR were performed using SYBR Green master mix using standard protocols. All qPCR primer sequences used are shown in Table S8, Supporting Information. *β*‐actin was used as an internal control.

### Protein Half‐Life Assay

After achieving ≈70% confluence, cells in 12‐well plates were transfected with the indicated plasmids using Lipofectamine 2000. 36 h later, the cells were treated with cycloheximide (CHX, 50 µg mL^−1^) for the indicated times before collection for immunoblot analysis.

### MTT Assay

For MTT assays, cells were seeded into 96‐well plates (10^3^ cells per well) and cultured in DMEM supplemented with 10% FBS. After 3–7 days of incubation, MTT was added and cells were incubated for 4 h at 37 °C. At the indicated time, this was replaced with 200 *µ*L of DMSO. Absorbance was then read at 570 nm using an ELX800 absorbance microplate reader.

### In Vitro Phosphorylation Assay

Recombinant GST‐*β*‐catenin‐His and PMVK‐His were prepared and purified from Rossita. 1 µg GST‐*β*‐catenin‐His protein was incubated with 1 µg PMVK‐His in a 50 *µ*L kinase reaction buffer (20 mm Tris‐HCl (pH 7.5), 10 mm MgCl_2_, 50 mm KCl, 30 *µ* m ATP) at 37 °C for 0.5 h. Reactions were stopped by the addition of SDS sample buffer, and samples were then heated for 5 min at 95 °C. Phosphorylation of *β*‐catenin Ser184 was identified by mass spectrometry and immunoblot with *β*‐catenin p‐Ser184 antibody that was generated by ABclonal by immunizing rabbits with a S184‐monophosphorylated peptide (KKEA(S‐pho)RHA) conjugated with keyhole limpet hemocyanin.

### In Vivo Ubiquitination Assay

36 h after transfection, cells were treated with 20 *µ* m MG132 for 6 h. They were then lysed in RIPA lysis buffer as described above. The samples were boiled for 10 min in SDS sample buffer and analyzed by immunoblot with the indicated antibodies.

### Microscale Thermophoresis

A Monolith NT.115 instrument (NanoTemper Technologies, Germany) was used to measure the *Kd* of binding of MAV‐5PP to CKI*α*‐GFP protein and PMVKi5 to PMVK‐GFP protein. All samples to be tested were first solubilized in RIPA buffer. The CKI*α*‐GFP or PMVK‐GFP was added to each dilution and incubated at room temperature for 5 min. The samples were loaded into silica capillaries (NanoTemper Technologies, Germany). The measurements were performed at 25 °C using 60% LED power and medium MST power. Data were analyzed with Nano‐Temper Analysis software, v.2.3.

### Serum Cholesterol, Triglyceride, ALT, and AST

Serum cholesterol, triglyceride, ALT, and AST were measured using colorimetric analysis kits (Elabscience, Wuhan). All experimental steps were performed according to the manufacturer's instructions.

### Sources of Human HCC Samples

Human HCC samples were used as previously described.^[^ ^34^ ^]^ All procedures that involved human HCC samples collection were approved by the ethics committee of Wuhan University (No.2022030) and the Union Hospital in Wuhan, China and conformed to the ethical guidelines of the 1975 Declaration of Helsinki. Informed written consent of all participants was signed by all individuals or their families and obtained at the Union Hospital in Wuhan, China. The diagnoses of all samples were confirmed by histological review.

### Docking and Molecular Modeling

The PMVK‐based crystal structure (PDB: 3CH4) was used for computer‐based virtual docking of 1.7 million small molecules from the ChemDiv database. Hydrogenation and energy optimization of database molecules relied on Schrodinger's Ligprep program. Pre‐processing of protein structures used Schrodinger's Protein Preparation program. Next, the HTVS, SP, and XP docking procedures of Schrodinger's Glide module were used to cascade docking of the database.^[^ ^37^ ^]^ Finally, the top 5 candidate small molecules were selected for cellular experiments. ChemBioDraw Ultra 17.0 was used to draw the structure of MVA‐5PP, the protein structures of the destruction complexes were all from the PDB database (APC PDB, 3NMZ; Axin1 PDB, 1DK8; *β*‐catenin PDB, 1QZ7; *β*‐TrCP PDB, 1P22; CKI*α* PDB, 6GZD; GSK3*β* PDB, 1I09; MVD PDB, 3D4J; PMVK PDB, 3CH4), and were docked using Discovery Studio.^[^ ^38^ ^]^

### Luminescent Kinase Assay

A Luminescent Kinase Assay Kit was used to quantify enzymatic activity of PMVK by quantifying ATP consumption. The kinase reaction system consisted of 50 mm HEPES (pH = 8.0), 50 mm KCl, 3 mm MgCl_2_, 200 *µ* m ATP, 40 *µ* m MVA‐5P, and 5 *µ* m PMVK, which was incubated at 37° for 5 min. Fluorescence intensity was measured by a luminometer. All experimental steps follow the manufacturer's instructions.

### Data Availability

High‐throughput sequencing results for protein class analysis and pathway enrichment analysis were analyzed in the websites (https://www.pantherdb.org). HCC datasets were downloaded from The Cancer Genome Atlas (TCGA) data portal (https://www.tcgadata.nci.nih.gov). PMVK mRNA levels were analyzed from TCGA and NCBI GEO databases (https://www.ncbi.nlm.nih.gov). Protein crystal structures were downloaded from PDB databases (https://www.pdbus.org). Small molecules were downloaded from the ChemDiv database (https://www.chemdiv.com).

### Statistics and Reproducibility

Data are presented as mean ± SD or mean ± SEM. Log‐rank tests were used for survival analyses. Student's *t*‐test was used for comparisons between two groups. One‐way ANOVA was used for multiple comparisons in patient populations. The correlation coefficient (*r*) and *p* values were obtained from Pearson correlation analysis, Pearson's *r* was calculated by GraphPad Prism 8. GraphPad Prism 8 was used for statistical calculations. *p* < 0.05 was considered to be statistically significant; *p < 0.05, **p < 0.01, ***p < 0.001, ****p < 0.0001; n.s., not significant. The data analyses were performed using unbiased software programs or algorithms. All experiments were repeated with a minimum of three independent repeats unless otherwise noted. Each experiment was repeated independently with similar results.

## Conflict of Interest

The authors declare no conflict of interest.

## Author Contributions

Z.C., F.W., and Y.L. designed the study; Z.C. performed most of the experiments; Y.T., M.L., and M.W. constructed plasmids; X.‐J.Z., Z.C., and F.W. performed to analyze sequencing and clinical data; J.Z., C.T., and X.‐Y.Z. performed some animal experiments. All authors discussed the results. Z.C., E.P., F.W., and Y.L. wrote the manuscript with comments from all authors.

## Supporting information

Supporting Information

## Data Availability

Research data are not shared.
