# Supplementary material for: Phosphomevalonate Kinase Controls β‐Catenin Signaling via the Metabolite 5‐Diphosphomevalonate
Source: Adv Sci (Weinh). 2023 Feb 21;10(12):2204909. doi: 10.1002/advs.202204909 (PMC10131864; doi:10.1002/advs.202204909)
Supplement: Supplementary file 1 — Supporting Information [file ADVS-10-2204909-s001.pdf]

ADVANCED SCIENCE
Open Access

Supporting Information

for *Adv. Sci.*, DOI 10.1002/advs.202204909

Phosphomevalonate Kinase Controls β-Catenin Signaling via the Metabolite 5-Diphosphomevalonate

Zhiqiang Chen, Xinyi Zhou, Xiaojun Zhou, Yi Tang, Mingzhu Lu, Jianhong Zhao, Chenhui Tian, Mingzhi Wu, Yanliang Liu, Edward V. Prochownik, Fubing Wang and Youjun Li**

Extended Data

Phosphomevalonate kinase controls β-catenin signaling via the metabolite 5-diphosphomevalonate

Extended Data Figure 1

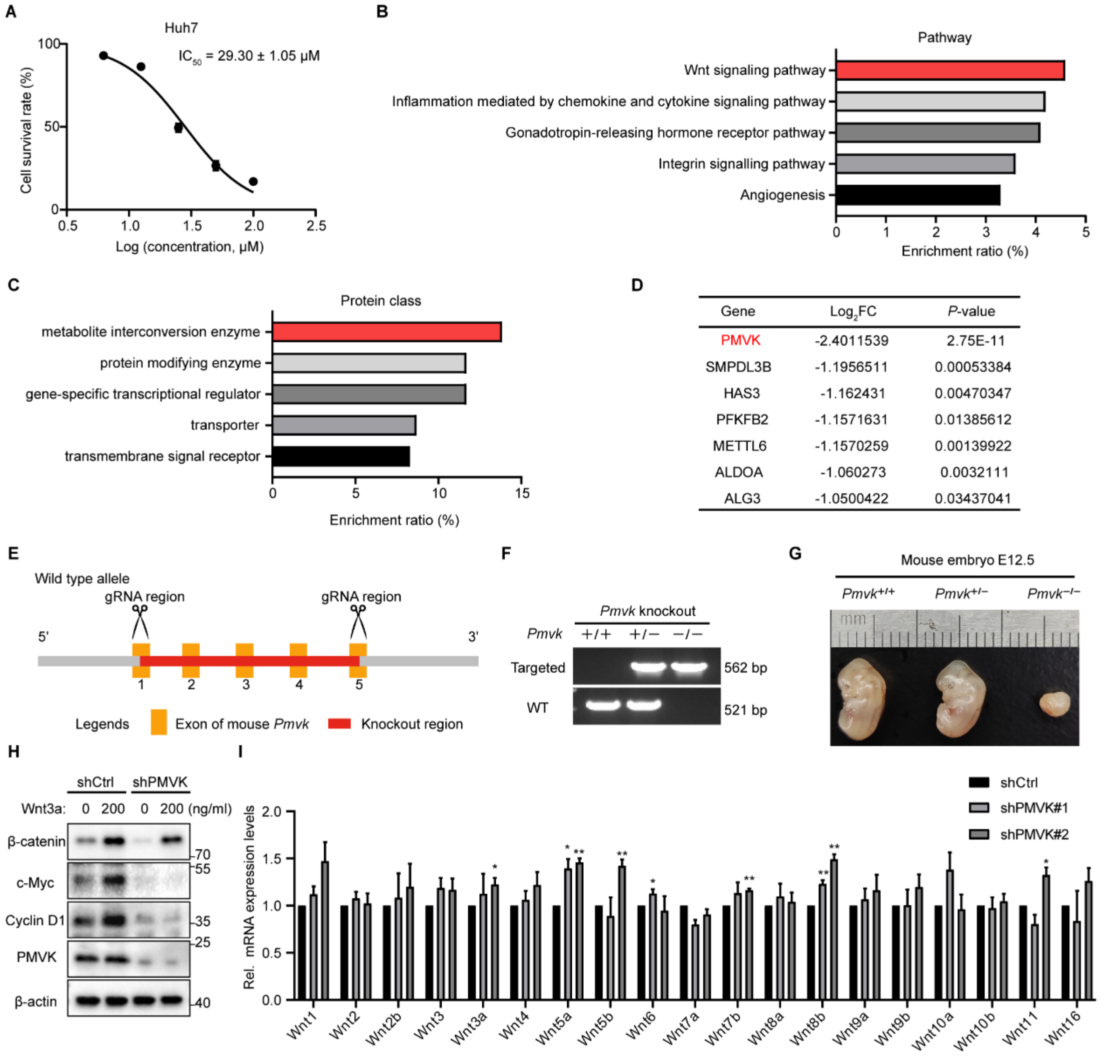

Extended Data Figure 1 CRISPR-cas9 library screens for PMVK stabilizing β-catenin protein in Huh7 cells.

(**A**) IC_{50} values of XAV939 were determined in Huh7 cell lines by MTT assay. Data are shown as mean ± SEM. (**B**) High throughput screening data by pathway enrichment analysis. (**C**) High throughput screening data by protein class enrichment analysis. (**D**) 7 candidate genes for β-catenin inhibitor resistance for Figure 1B. Log_2FC, Log_2(Foldchange). (**E**) Schematic diagram of the construction of *Pmvk* knockout mice. (**F**) Detection of mouse genotype by agarose gel electrophoresis. (**G**) Images were extracted from wild-type (WT), *Pmvk*$^{+/-}$ and *Pmvk*$^{-/-}$ E12.5 embryos. (**H**) Western blot analysis of the protein levels of β-catenin and its downstream target genes in wild-type and PMVK knockdown Huh7 cells treated using Wnt3a (200 ng/mL) for 6 h. (**I**) qRT-

PCR analysis was performed to measure the mRNA levels of Wnt family in Huh7 cells. The *P* values were determined by unpaired two-sided Student's t-test.

Extended Data Figure 2

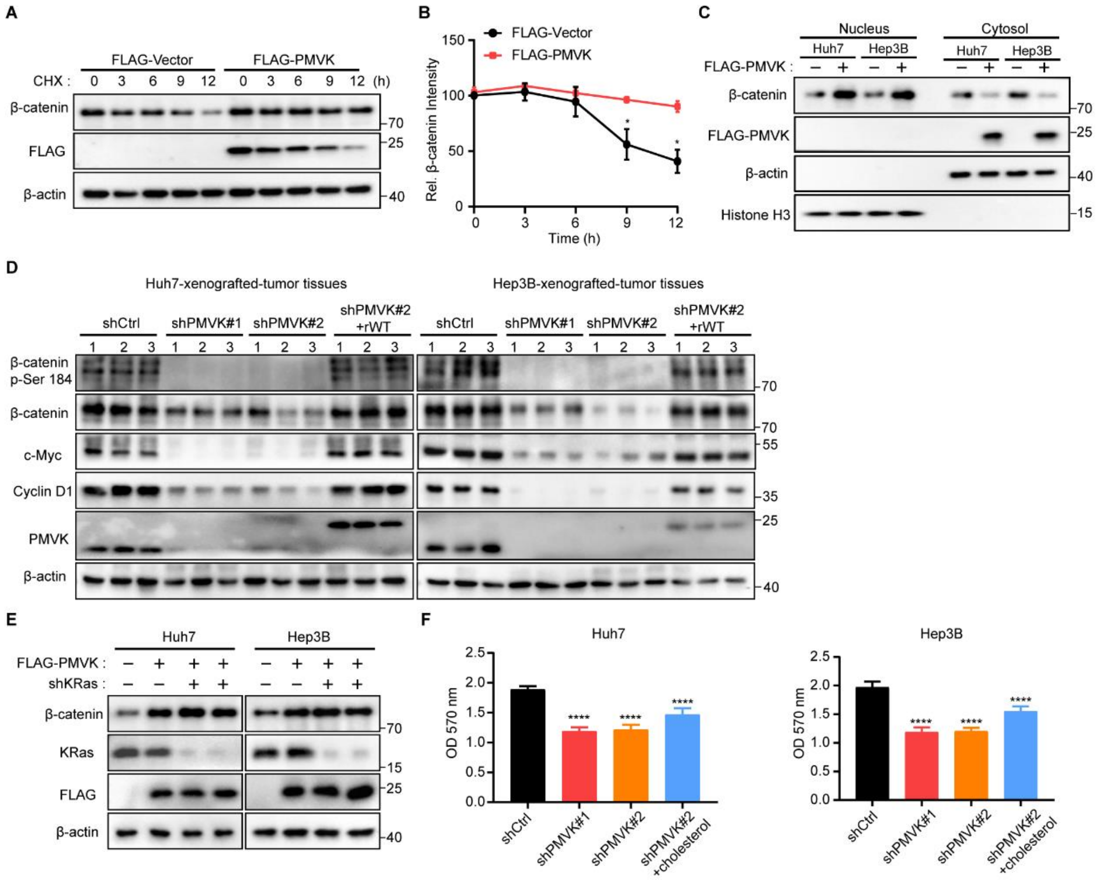

Extended Data Figure 2 Overexpression of PMVK increases β-catenin half-life and promotes nuclear localization.

(**A**) HEK293 cells were transfected with the indicated vectors. Cells were treated with CHX (50 μg/ml) for the indicated time and the expression of FLAG-PMVK and β-catenin were analyzed by western blotting. (**B**) The intensity of β-catenin expression for each time point in (A) was quantified by densitometry, with β-actin as a normalizer. (**C**) Distribution of FLAG-PMVK and β-catenin protein levels in the nucleus or cytoplasm in the indicated cell lines. β-actin as a cytoplasm normalizer and Histone H3 as a nucleus normalizer. (**D**) Western blot analysis of the protein levels of β-catenin (total and p-S184 form) and its downstream target genes in xenografted-tumor tissues. (**E**) Huh7 and Hep3B cells were transfected with the indicated vectors and the expression of β-catenin was analyzed by western blotting. (**F**) Cell proliferation assays were performed in Huh7 (left) and Hep3B (right) cells stably expressing the indicated plasmids. 0.5 μg/ml of cholesterol was supplemented in PMVK knockdown cell lines

and cell viability was assayed after 7 days. Data are shown as mean ± SD. The *P* values were determined by unpaired paired two-tailed Student's t-test.

Extended Data Figure 3

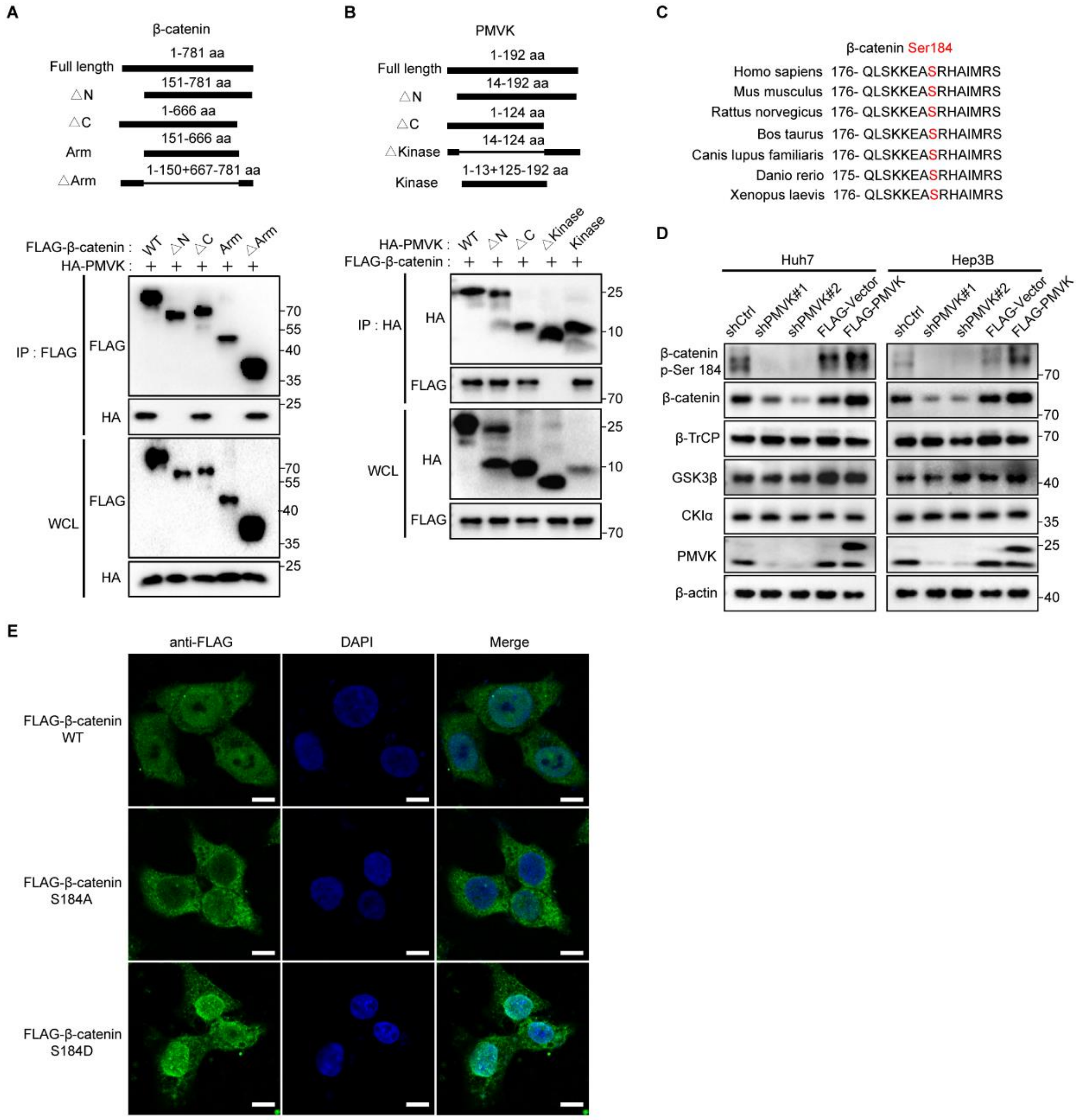

Extended Data Figure 3 PMVK interacts with β-catenin.

(**A**) Schematic diagram showed the structure of β-catenin and truncation mutants used. FLAG-β-catenin WT or truncation mutants were co-expressed with HA-PMVK in HEK293. Extracts were immunoprecipitated with anti-FLAG affinity agarose and examined by Western blotting. WCL, Whole cell lysate. (**B**) HA-PMVK WT or truncation mutants were co-expressed with FLAG-β-catenin in HEK293. Extracts were immunoprecipitated with anti-HA magnetic beads and examined by Western blotting. (**C**) Alignment of S184 and adjacent amino acid sequence of β-catenin among different species. (**D**) Western blot analysis of the protein levels of β-catenin in Huh7 and Hep3B cells transfected with the indicated vectors. (**E**) HEK293 cells were transfected with the

indicated plasmids 48 h, fixed and immunostained with anti-FLAG before confocal microscopy. Scale bar, 10 μm. green, anti-FLAG; blue, DAPI.

Extended Data Figure 4

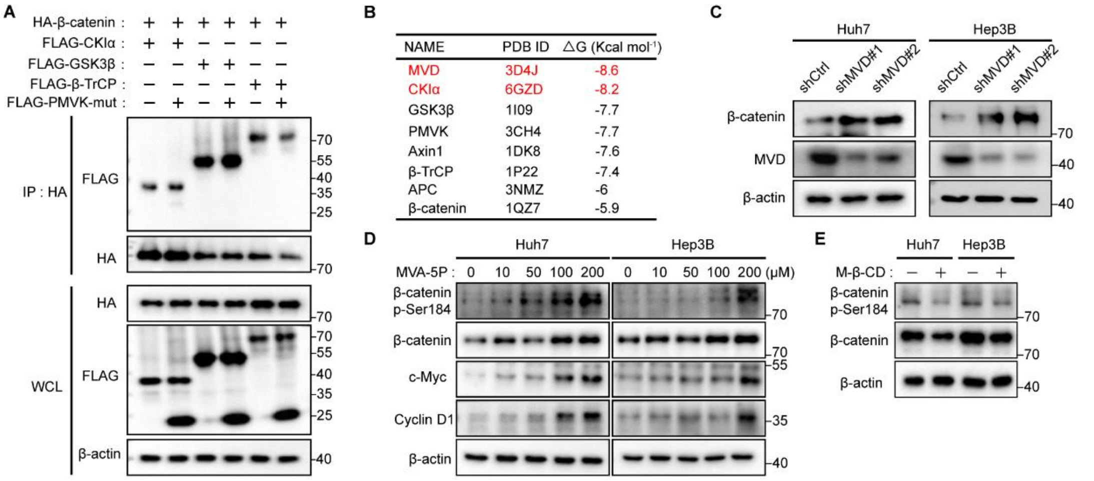

Extended Data Figure 4 MVA-5pp, not PMVK, competitively binds CK1α to stabilize β-catenin.

(**A**) HEK293 cells were transfected with the indicated plasmids and then lysed in RIPA buffer. Immuno-precipitation of HA-β-catenin proteins with anti-HA magnetic beads and subjected to immunoblot assay. FLAG-PMVK-mut (K17A, S20A, G21A, K22A and D23A). (**B**) Computerized virtual docking of MVA-5PP and disruption complex-associated proteins were based on protein crystal structures from PDB. (**C**) β-catenin and MVD protein expression in the indicated MVD-knockdown and control cancer cell lines. (**D**) Huh7 and Hep3B cells were treated with MVA-5P for 24 h and subjected to immunoblot assay of β-catenin, c-Myc and Cyclin D1 protein levels. (**E**) HCC cell lines were treated with Methyl-β-cyclodextrin (2.5 mM) for 24 h in and the protein levels of β-catenin (total and p-S184 form) were detected using western blot.

Extended Data Figure 5

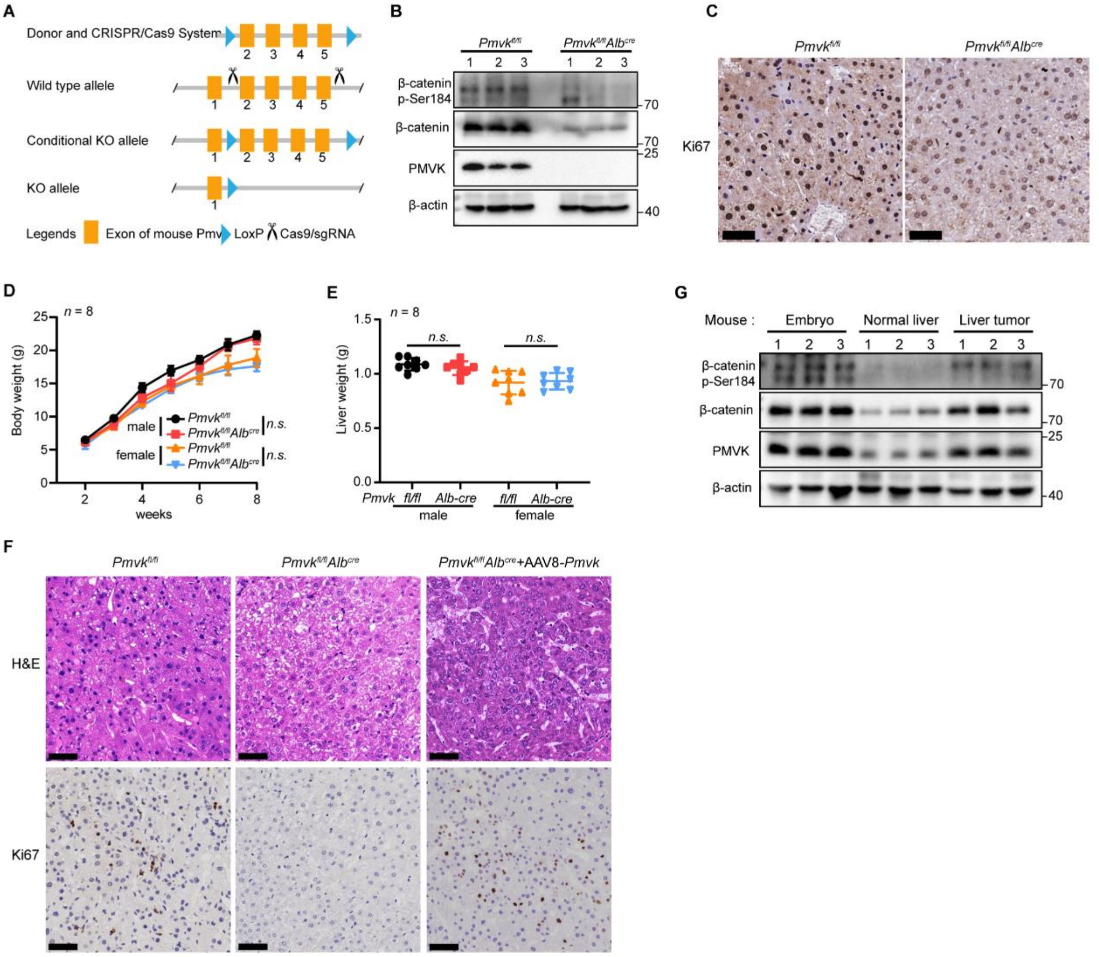

Extended Data Figure 5 Liver-specific deletion of PMVK inhibits HCC progression in mice.

(**A**) Schematic diagram of the construction of Liver-specific deletion of *Pmvk* knockout mice. (**B**) β-catenin, β-catenin p-Ser184 and PMVK protein levels in normal liver tissue of $Pmvk^{fl/fl}$ and $Pmvk^{fl/fl}Alb^{cre}$ mice. (**C**) Ki67 staining showed proliferative capacity of normal liver tissue in 8-week-old $Pmvk^{fl/fl}$ and $Pmvk^{fl/fl}Alb^{cre}$ male mice. Scale bar, 1 mm or 50 μm. (**D**) Body weight changes in $Pmvk^{fl/fl}$ and $Pmvk^{fl/fl}Alb^{cre}$ mice. (**E**) Liver weight of eight-week-old in $Pmvk^{fl/fl}$ and $Pmvk^{fl/fl}Alb^{cre}$ mice. (**F**) Representative IHC images of H&E and Ki67 in tumor tissues for Figure 5b. Scale bar, 50 μm. (**G**) Western blot analysis of protein levels of β-catenin (total and p-S184 forms) and PMVK in liver tissues from mouse embryonic (E12.5), adult wild-type mice and HCC model mice. Data are shown as mean ± SD. The *P* values were determined by unpaired two-sided Student's t-test.

Extended Data Figure 6

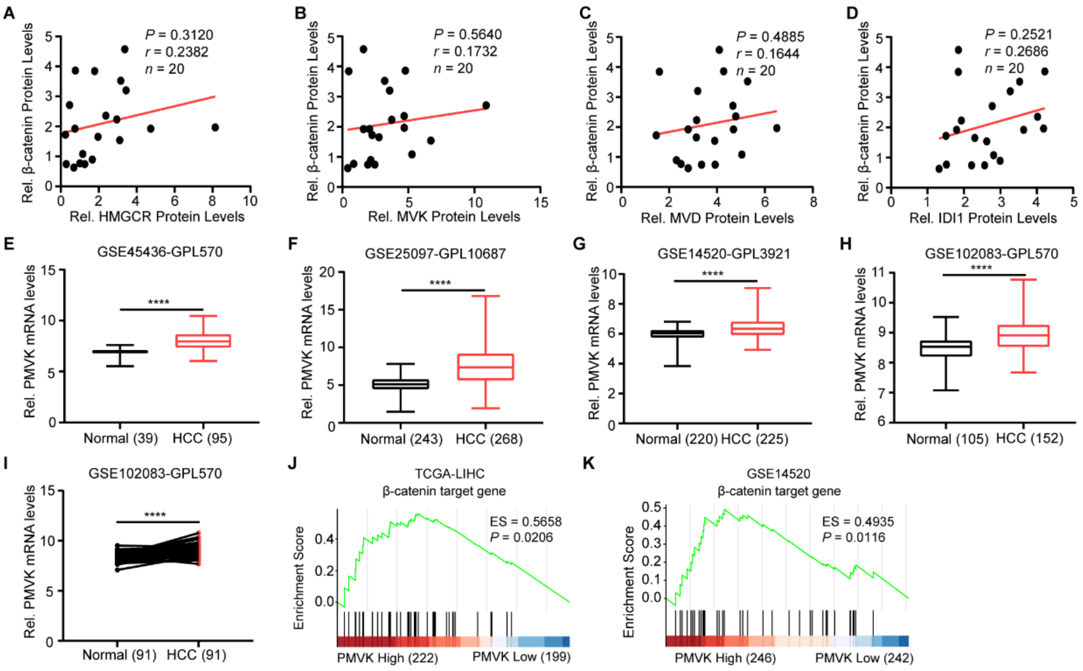

L

Variable	High (N=183)	Low (N=182)	*P*-value	Variable	High (N=183)	Low (N=182)	*P*-value
Gender			0.02	Histologic grade			0.83
Female	49 (13.42%)	70 (19.18%)		G1	28 (7.78%)	27 (7.50%)	
Male	134 (36.71%)	112 (30.68%)		G2	91 (25.28%)	84 (23.33%)	
Median age			0.37	G3	56 (15.56%)	62 (17.22%)	
≥61 years	100 (27.40%)	90 (24.66%)		G4	7 (1.94%)	5 (1.39%)	
<61 years	83 (22.74%)	92 (25.21%)		pT status			0.21
Survival status			0.16	T1	99 (27.35%)	81 (22.38%)	
Alive	124 (34.07%)	110 (30.22%)		T2	44 (12.15%)	47 (12.98%)	
Dead	58 (15.93%)	72 (19.78%)		T3	32 (8.84%)	46 (12.71%)	
pM status			0.27	T4	7 (1.93%)	6 (1.66%)	
M0	134 (50.38%)	129 (48.50%)		Clinical stage			0.04
M1	3 (1.13%)	0 (0.0+0%)		stage I	94 (27.57%)	76 (22.29%)	
pN status			0.14	stage II	44 (12.90%)	40 (11.73%)	
N0	124 (49.21%)	124 (49.21%)		stage III	31 (9.09%)	52 (15.25%)	
N1	0 (0.0+0%)	4 (1.59%)		stage IV	3 (0.88%)	1 (0.29%)	

Extended Data Figure 6 PMVK expression is increased in human HCC and correlates with poor survival.

(**A-D**) Correlation of HMGCR (A), MVK (B), MVD (C) and IDI1 (D) protein levels in HCC tissues for Figure 6A. Each point is an individual sample. (**E-H**) Relative PMVK mRNA levels in normal liver and HCCs from GSE45436 (E), GSE25097 (F), GSE14520 (G) and GSE102083 (H). (**I**) PMVK mRNA levels were analyzed in paired HCC tissues from GSE102083. (**J** and **K**) Gene-set enrichment analysis (GSEA) of PMVK in HCC patients from TCGA-LIHC and GSE14520. Representative GSEA plots indicated that predefined gene sets involved in the β-catenin target genes were positively associated with high PMVK expression. ES enrichment score. (**L**) Correlation between Expression of PMVK and Clinicopathological Features of HCC

Patients. Data are shown as mean ± SD. The correlation coefficient (*r*) and *P* values in (A-D) were determined using two-tailed Pearson correlation analysis. The *P* values were determined by paired two-tailed Student's t-test for (I) and unpaired two-tailed Student's t-test for (E-H). Two-way analysis of variance (ANOVA) for (L).

Extended Data Figure 7

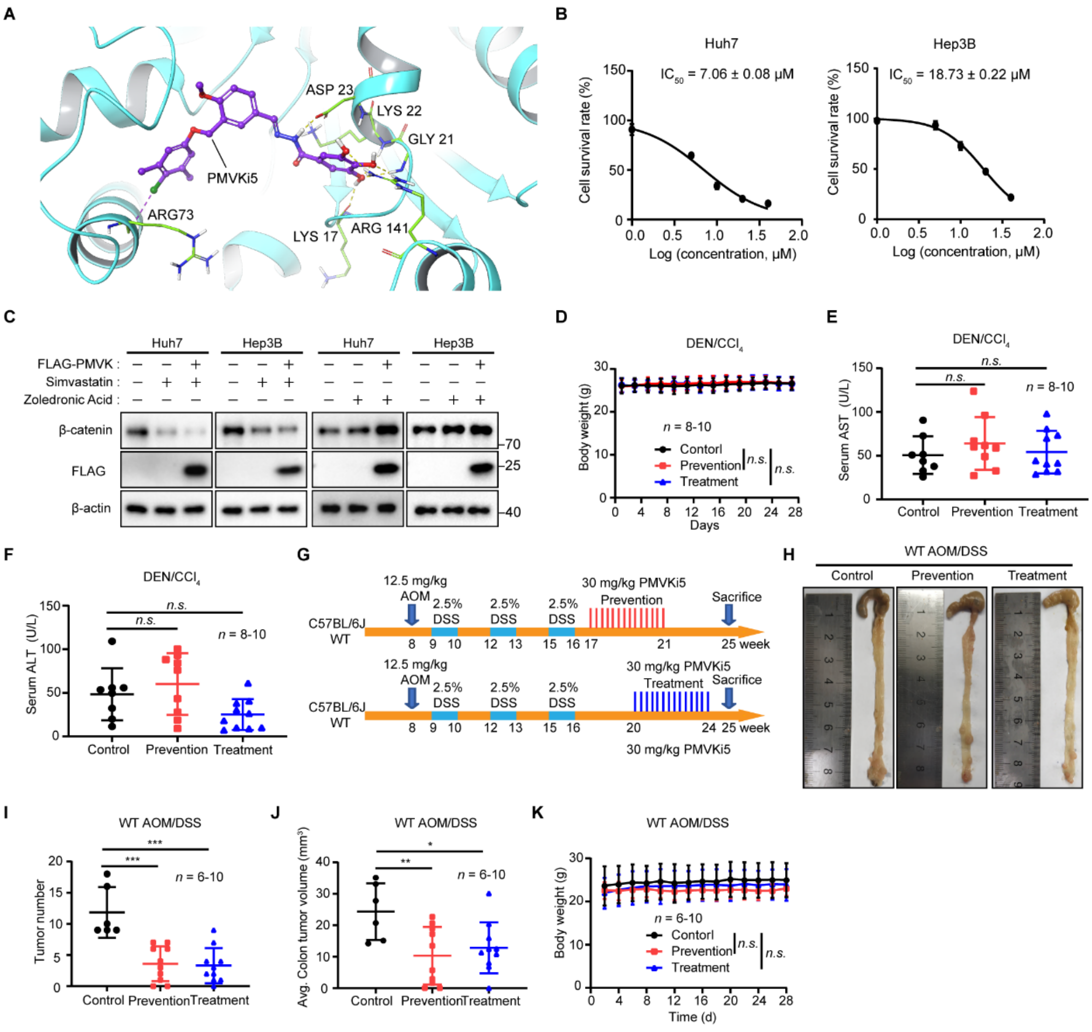

Extended Data Figure 7 PMVK inhibitor PMVKi5 blocks colorectal cancer tumor growth in vivo.

(**A**) The molecular docking of PMVKi5 and PMVK is shown based on the crystal structure of PMVK (PDB code: 3CH4), and the bound residues are including Lys17, Ser20, Gly21, Lys22, Asp23, Arg73 and Arg141. (**B**) IC_{50} values of PMVKi5 were determined in Huh7 and Hep3B cell lines by MTT assay. Data are shown as mean ± SEM. (**C**) Treatment with 10 μM simvastatin or 10 μM zoledronic acid for 24 h in wild-type and PMVK overexpressing HCC cell lines and immunoblotting for β-catenin protein levels. (**D**) Body weight was measured after implantation in the DEN/CCl_4 mouse model for Figure 7G. *n* = 8-10 per group. (**E**) Serum AST levels in mice from Figure 7G. (**F**) Serum ALT levels in mice from Figure 7G. (**G**) The schematic overview

of AOM/DSS-induced CRC mice model. AOM (12.5 mg/kg) was injected 8 weeks after birth. From 9, 12 and 15 weeks, add 2.5% DSS to the drinking water of mice for one week, from 17-21 weeks mice were intraperitoneal injected with 30 mg/kg PMVKi5 every two days for a total of 14 doses for prevention. Alternatively, from 20-24 weeks mice were treated in the same manner for treatment. (**H**) Colon images extracted from the indicated mice. (**I**) Tumor number of each colon for (H). (**J**) Average tumor volume of each colon for (H). (**K**) Body weight measured after implantation in the AOM/DSS mouse model. Data are shown as mean ± SD. The *P* values were determined by unpaired two-tailed Student’s t-test.

Supplementary Table 3. shRNA sequences.

shRNA	Target Sequences (5'>3')
shPMVK#1	GGAAGATTGTGGAGGGCATCT
shPMVK#2	GGAAGACAGCCATGCTCTTCA
shMVD#1	GAATGGAGACACGTTTCTGAA
shMVD#2	CCATCTCTTACCTCAATGCCA
shKRas#1	CAGTTGAGACCTTCTAATTGG
shKRas#2	GAGGGCTTTCTTTGTGTATTT

Supplementary Table 4. Primers for constructs.

Insert	Forward Primer (5'>3')	Reverse Primer (5'>3')
FLAG-PMVK	CGCGCGGCCGCATGGCCCCGCTGGGAGGC	CGCGTCGACAAGTCTGGAGCGGATAAATTCTATC
HA-PMVK-WT	CGCGAATTCGGATGGCCCCGCTGGGAGGC	CGCCTCGAGCTAAAGTCTGGAGCGGATAAATTC
HA-PMVK-△N	CGCGGATCCTTCAGCGGCAAGAGGAAATCC	CGCCTCGAGCTAAAGTCTGGAGCGGATAAATTC
HA-PMVK-△C	CGCGAATTCGGATGGCCCCGCTGGGAGGC	CGCCTCGAGCTAGGCCCCATAGGCCTCCC
HA-PMVK-△Kinase	CGCGGATCCATGGCCCCGCTGGGAGGCGCCCCGCGGCTGGTACTGCTGGTGACGCAGACGGTCCGC	CGCCTCGAGCTAAAGTCTGGAGCGGATAAATTC
HA-PMVK-Kinase	CGCGGATCCTTCAGCGGCAAGAGGAAATCC	CGCCTCGAGCTAGGCCCCATAGGCCTCCC
pET28-PMVK-His	CGCGGATCCATGGCCCCGCTGGGAGGC	CGCGAATTCTCAGTGGTGGTGGTGGTGGTGAAGTCTGGAGCGGATAAATTC
GST-β-Catenin-His	GATCTGGTTCCGCGTGGATCCATGGCTACTCAAGCTGATTTGATGG	ATGATGATGCGGCCGCTCGAGCAGGTCAGTATCAAACCAGGC
HA-β-Catenin WT	CCGAATTCGGTCGACCATGGCTACTCAAGCTGATTTG	GATCCCCGCGGCCGCTTACAGGTCAGTATCAAACCAGGC
HA-β-Catenin S184A	AGGAAGCTGCCAGACACGCTATC	GATAGCGTGTCTGGCAGCTTCCT
HA-β-Catenin S184D	AGGAAGCTGACAGACACGCTATC	GATAGCGTGTCTGTCAGCTTCCT
Flag-β-Catenin-WT	CGCGCGGCCGCATGGCTACTCAAGCTGATTTGATGG	CGCGTCGACCAGGTCAGTATCAAACCAGGC
Flag-β-Catenin-△N	CGCGCGGCCGCATGCGTGCAATCCCTGAACTGAC	CGCGTCGACCAGGTCAGTATCAAACCAGGC
Flag-β-Catenin-△C	CGCGCGGCCGCATGGCTACTCAAGCTGATTTGATGG	CGCGTCGACCTTGTCCTCAGACATTCGGAAC
Flag-β-Catenin-Arm	CGCGCGGCCGCATGCGTGCAATCCCTGAACTGAC	CGCGTCGACCTTGTCCTCAGACATTCGGAAC
Flag-β-Catenin-△Arm	ATGATGCAGAACTTGCCACACCACAAGATTACAAGAAACGGC	CGTTTCTTGTAATCTTGTGGTGTGGCAAGTTCTGCATCATC

FLAG-CK1α	CGCGCGGCCGCATGGCGAGTAGCAGCGGCTC	CGCGTCGACGAAACCTTTCATGTTACTCTTGG
FLAG-GSK3β	CGCGCGGCCGCATGTCAGGGCGGCCCAGAAC	CGCGTCGACGGTGGAGTTGGAAGCTGATGC
FLAG-β-trcp	CGCGCGGCCGCATGGACCCGGCCGAGGCGGTG	CGCGTCGACTCTGGAGATGTAGGTGTATGTTCGAG
PMVK-GFP	CGACCTCTCTCCCCAGGGGGATCCATGTACCCATACGATGTTCCAG	CTTGCTCACCATGGTGGCGGATCCAAGTCTGGAGCGGATAAATTCTATC
CK1α-GFP	CGACCTCTCTCCCCAGGGGGATCCATGTACCCATACGATGTTCCAG	CTTGCTCACCATGGTGGCGGATCCGAAACCTTTCATGTTACTCTTGG

Supplementary Table 5. Chemical reagent or kit.

Reagent or kit	Source	Identifier
XAV-939	Selleck	S1180
Cycloheximide	MCE	HY-12320
Methyl-β-cyclodextrin	MCE	HY-101461
Simvastatin	TargetMol	T0687
Zoledronic acid	TargetMol	T6739
MVA-5PP	Sigma-Aldrich	77631
MVA-5P	Sigma-Aldrich	7841
MG132	MCE	HY-13259
PMVKi5	ChemDiv	K087-0251
DEN	Sigma-Aldrich	N0258
AOM	Sigma-Aldrich	A5486
DSS	MP Biomedicals	02160110-CF
Trizol	Invitrogen	10296010
SYBR Green master mix	Abclonal	RK21203
TC	Elabscicence	E-BC-K109-M
TG	Elabscicence	E-BC-K261-M
AST	Elabscicence	E-BC-K236-M
ALT	Elabscicence	E-BC-K235-M
MTT	Sigma-Aldrich	M5655
HiScript III 1st Strand cDNA Synthesis Kit	Vazyme	R312-01
Kinase-Lumi™ Max Luminescent Kinase Assay Kit	Beyotime	S0158S

Supplementary Table 6. Antibodies used in this study.

Antibodise	Source	Identifier
Ki67	Abcam	ab16667
PMVK	Proteintech	15674-1-AP
PMVK(H-9)	Santa Cruz	SC-390775
β-catenin	Proteintech	51067-2-AP
β-catenin p-Ser184	Abclonal	NA
FLAG	Proteintech	25043-1-AP
HA	Abclonal	AE008
Histone H3	Proteintech	17168-1-AP
c-Myc	Santa Cruz	SC-764
Cyclin D1	Proteintech	60686-1-Ig
β-actin	Proteintech	66009-1-Ig
Myc-tag	Proteintech	16286-1-AP
CK1α	Abclonal	A9308
β-Catenin p-Thr 41/Ser 45	Sigma-Aldrich	SAB4504349
His-tag	Proteintech	66005-1-Ig
HMGCR	Abclonal	A1633
MVK	Proteintech	12228-1-AP
MVD	Abclonal	A15240
IDI1	Abclonal	A13826
β-TrCP	Abclonal	A21951
GSK3β	Abclonal	A2081
KRas	Abclonal	A1190
ABflo™ 488-conjugated Goat Anti-Rabbit IgG	Abclonal	AS053
Goat Anti-Rabbit IgG (H+L)	Jackson	JAC-111-035-003

Goat Anti-<br>Mouse IgG (H+L)	Jackson	JAC-115-035-003
Goat Anti-Mouse IgG<br>Heavy Chain	Abclonal	AS064
Goat Anti-Mouse IgG<br>Light Chain	Abclonal	AS062

Supplemental Table 7. Mouse genotypes primers.

Gene	Forward Primer (5'>3')	Reverse Primer (5'>3')
PMVK KO WT	TGTGTAGAAGGGAACTTGAGGCT	CTCCATAAAGCCCTCCATCACTT
PMVK KO Target	CTTAGATACTAGGCTGCAAGGCT	CCCACTTGTAACACAGAGACCT
Pmvk$^{fl/fl}$	GGGAGCACCTATTTCATAGCACA	CATCATCTGCCCTACCGTCACAC
Alb-cre	CCTGTTACGTATAGCCGAAA	CTTAGCGCCGTAAATCAATC

Supplemental Table 8. qPCR primers.

Gene	Forward Primer (5'>3')	Reverse Primer (5'>3')
PMVK	CCTTTCGGAAGGACATGATCC	TCTCCGTGTGTCACTCACCA
CTNNB1	CATCTACACAGTTTGATGCTGC	GCAGTTTTGTCAGTTCAGGGA
MMP7	GAGTGAGCTACAGTGGGAACA	CTATGACGCGGGAGTTTAACAT
VEGF	AGGGCAGAATCATCACGAAG	AGGGTCTCGATTGGATGGCA
CD44	CTGCCGCTTTGCAGGTGTA	CATTGTGGGCAAGGTGCTATT
AXIN2	AGCCAAAGCGATCTACAAAAGG	AAGTCAAAAACATCTGGTAGGC
CCND1	CAATGACCCCGCACGATTTC	CATGGAGGGCGGATTGGAA
MYC	GGCTCCTGGCAAAAGGTCA	CTGCGTAGTTGTGCTGATGT
Wnt1	CGATGGTGGGGTATTGTGAAC	CCGGATTTTGGCGTATCAGAC
Wnt2	CCGAGGTCAACTCTTCATGGT	CCTGGCACATTATCGCACAT
Wnt2b	GGGGCACGAGTGATCTGTG	GCATGATGTCTGGGTAACGCT
Wnt3	AGGGCACCTCCACCATTTG	GACACTAACACGCCGAAGTCA
Wnt3a	AGCTACCCGATCTGGTGGTC	CAAACTCGATGTCCTCGCTAC
Wnt4	CTCCACACTCGACTCCTTGC	CCGAAGAGATGGCGTACACG
Wnt5a	TCGACTATGGCTACCGCTTTG	CACTCTCGTAGGAGCCCTTG
Wnt5b	CATGGCCTACATAGGGGAGG	CTGTGCTGCAATTCCACCG
Wnt6	GGCAGCCCCTTGGTTATGG	CTCAGCCTGGCACAACTCG
Wnt7a	CTGTGGCTGCGACAAAGAGAA	GCCGTGGCACTTACATTCC
Wnt7b	CGCAGCTATCAGAAGCCCAT	CAGGTGTTGCACTTGACGA
Wnt8a	CAGAGGCGGAACTGATCTTTT	CACCTGGATGTGTTGTGGC
Wnt8b	AAGGCCGAGAGTGCCTAAG	CTGCGCGGCTACAGAAGTA
Wnt9a	CCACCGTGAGAAGAACTGC	GCCTGCACTCCACATAGCA
Wnt9b	TGTGCGGTGACAACCTCAAG	ACAGGAGCCTGATACGCCAT
Wnt10a	GGTCAGCACCCAATGACATTC	TGGATGGCGATCTGGATGC
Wnt10b	GTGAGCGAGACCCCACTATG	CACTCTGTAACCTTGCACTCATC

Wnt11	GGAGTCGGCCTTCGTGTATG	GCCCGTAGCTGAGGTTGTC
Wnt16	TTCAGACACGAGAGATGGAACT	CCAGCCTTCACTTGCTGAG
ACTB	ATCATGAAGTGTGACGTGGACAT	AGGAGCAATGATCTTGATCTTCA
